# Diagnostic Performance of Comprehensive Point‐of‐Care Ultrasound for Pediatric Tuberculosis in Spain: A Prospective Observational Study

**DOI:** 10.1002/jcu.70228

**Published:** 2026-03-15

**Authors:** Isabelle Munyangaju, Antoni Noguera‐Julian, Aleix Soler‐Garcia, Antoni Soriano‐Arandes, José Miguel Escudero Fernández, Maria Espiau, Begoña Santiago Garcia, Alicia Hernanz‐Lobo, Ángel M. Lancharro Zapata, Enrique Ladera, Angela Manzanares, Daniel Blazquez, Elisa Aguirre Pascual, Isabelle Thierry‐Chef, Danilo Buonsenso, Xavier Serres‐Créixams, Quique Bassat, Elisa Lopez‐Varela

**Affiliations:** ^1^ Barcelona Institute for Global Health ‐ Hospital Clínic Universitat de Barcelona Barcelona Spain; ^2^ Facultat de Medicina i Ciències de la Salut Universitat de Barcelona (UB) Barcelona Spain; ^3^ Nucleo de Pesquisa Pediatria, Faculdade de Medicina Universidade Eduardo Mondlane Maputo Mozambique; ^4^ Malalties Infeccioses i Resposta Inflamatòria Sistèmica en Pediatria, Servei de Malalties Infeccioses i Patologia Importada Institut de Recerca Pediàtrica Sant Joan de Déu Barcelona Spain; ^5^ Centro de Investigación Biomédica en Red de Epidemiología y Salud Pública (CIBERESP) Madrid Spain; ^6^ Departament de Cirurgia i Especialitats Medicoquirúrgiques, Facultat de Medicina i Ciències de la Salut Universitat de Barcelona Barcelona Spain; ^7^ Red de Investigación Translacional en Infectología Pediátrica RITIP Madrid Spain; ^8^ Unitat de Patologia Infecciosa i Immunodeficiències de Pediatria (UPIIP) Hospital Universitari Vall d’Hebron Barcelona Spain; ^9^ Vall d’Hebron Research Institute Infection and Immunity in Children Barcelona Spain; ^10^ Radiologia Pediàtrica Hospital Universitari Vall d’Hebron Barcelona Spain; ^11^ Pediatric Infectious Diseases Department Gregorio Marañón University Hospital Madrid Spain; ^12^ Gregorio Marañón Health Research Institute (IiSGM) Madrid Spain; ^13^ Centro de Investigación Biomédica en Red de Enfermedades Infecciosas (CIBERINFEC) Instituto de Salud Carlos III Madrid Spain; ^14^ Pediatric Radiology Gregorio Marañon University General Hospital Madrid Spain; ^15^ Radiologia Pediàtrica Hospital Sant Joan de Déu Barcelona Spain; ^16^ Pediatric Infectious Diseases Unit, Hospital Universitario 12 de Octubre Instituto de Investigación Hospital 12 de Octubre, Universidad Complutense Madrid Spain; ^17^ Radiologia Pediàtrica Hospital Universitario 12 de Octubre Madrid Spain; ^18^ CIBER Epidemiología y Salud Pública (CIBERESP) Av. Monforte de Lemos Madrid Spain; ^19^ Universitat Pompeu Fabra (UPF) Barcelona Spain; ^20^ The French Authority for Nuclear Safety and Radiation Protection Fontenay‐aux‐Roses cedex France; ^21^ Department of Woman and Child Health and Public Health Fondazione Policlinico Universitario A. Gemelli IRCCS Rome Italy; ^22^ Area Pediatrica, Dipartimento di Scienze della Vita a Sanità Pubblica Università Cattolica del Sacro Cuore Rome Italy; ^23^ Global Health Research Institute, Istituto di Igiene Università Cattolica del Sacro Cuore Roma Italy; ^24^ Radiodiagnóstico, Ecografia intervencionista Hospital Universitario Vall d’Hebron Barcelona Spain; ^25^ Centro de Investigação em Saúde de Manhiça (CISM) Manhiça Mozambique; ^26^ ICREA Barcelona Spain; ^27^ Departament de Pediatria, Hospital Sant Joan de Déu Universitat de Barcelona Barcelona Spain

**Keywords:** diagnostic imaging, mediastinal lymphadenopathy, pediatric tuberculosis, point‐of‐care ultrasound

## Abstract

**Background:**

Diagnosing tuberculosis (TB) in children is challenging because of nonspecific symptoms, low microbiological yield, and imaging limitations. Comprehensive point‐of‐care ultrasound (cPOCUS) may detect mediastinal lymphadenopathy, but evidence remains limited.

**Methods:**

We conducted a prospective observational study in four tertiary hospitals in Spain (May 2023–June 2024) enrolling children (0–18 years) with presumptive TB. All participants underwent chest X‐ray (CXR) and cPOCUS; chest CT was performed when clinically indicated. cPOCUS assessed lung, mediastinal, and abdominal compartments using a standardized protocol. Images were independently reviewed by expert readers blinded to clinical data. Diagnostic performance was evaluated against a final TB classification based on clinical, immunological, microbiological, and conventional radiological findings, excluding cPOCUS.

**Results:**

Twenty‐seven children were included (median age 9.0 years), of whom 12 (44.4%) had TB disease. CT was performed in 14 (51.9%). Only 22.2% of cPOCUS examinations were complete. Mediastinal lymphadenopathy was the most frequent abnormality. cPOCUS detected abnormalities in 58.3%–63.6% of TB cases, particularly aortopulmonary window lymph nodes and small subpleural consolidations, with moderate sensitivity and high specificity (80.0%–86.7%). Inter‐reader agreement was high (*κ* = 0.92).

**Conclusions:**

In this pilot prospective study, cPOCUS demonstrated limited diagnostic performance but high inter‐reader reliability. These exploratory findings suggest that cPOCUS may complement standard imaging for selected abnormalities (particularly aortopulmonary window lymphadenopathy), but larger studies with optimized protocols are needed before broader clinical implementation.

## Graphical abstract

In children with presumptive tuberculosis, comprehensive point‐of‐care (cPOCUS) identified key thoracic abnormalities with high specificity and excellent inter‐reader reliability. Although diagnostic sensitivity was modest and examinations often incomplete, cPOCUS shows promise as a complementary, radiation‐free imaging tool.

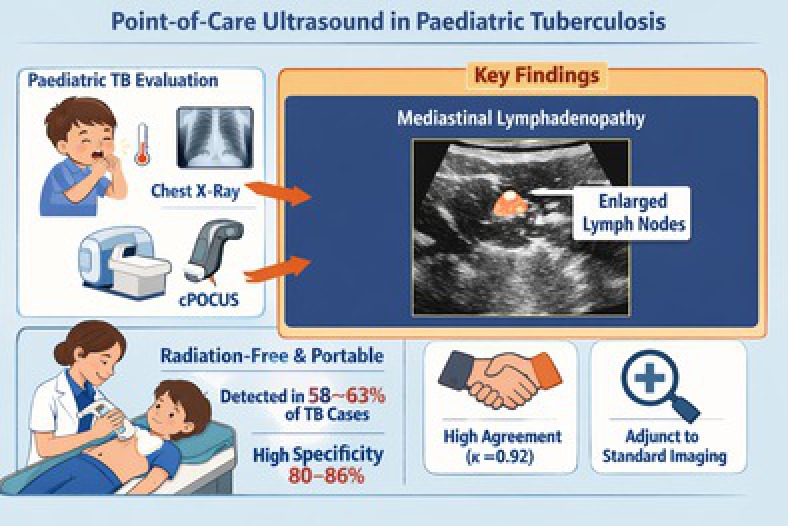

**Abbreviations**
AOP: aortopulmonary
cPOCUS: comprehensive point‐of‐care ultrasound
CT: computed tomography
CXR: chest X‐ray
HIV: human immunodeficiency virus
IGRA: interferon‐gamma release assay
SOP: standard operating procedure
TB: tuberculosis
TST: tuberculin skin test

## 1 Introduction

Tuberculosis (TB) remains a major cause of childhood morbidity and mortality globally, with an estimated 1.2 million children developing the disease in 2024 (WHO 2025). Despite advances in molecular diagnostics and preventive therapy, pediatric TB continues to be underdiagnosed and undertreated, especially in young children who often present with non‐specific clinical features and low bacillary loads. Conversely, the opposite scenario is also common; many children with ambiguous clinical presentations and overlapping symptoms are started on TB treatment despite lacking definitive evidence of the disease, resulting in unnecessary and prolonged therapy. The challenge is compounded by the limitations of existing diagnostic tools: sputum‐based tests have shown suboptimal sensitivity in children, and radiological modalities such as chest X‐ray (CXR) or computed tomography (CT) may be inaccessible in high burden settings due to high costs (Basile et al. 2022; WHO 2023; Kendall 2017; Reuter et al. 2019; Basu and Chakraborty 2025; Moore et al. 2024). These challenges highlight the need for better diagnostic tools to accurately identify children with true TB disease.

Point‐of‐care ultrasound (POCUS) is emerging as an affordable, radiation‐free, and portable imaging modality in the evaluation of infectious diseases. In adults with pulmonary and extrapulmonary TB, ultrasound can identify characteristic findings such as pleural effusion, pericardial fluid, ascites, or abdominal lymphadenopathy (Díez‐Vidal et al. 2024; Rea et al. 2021; Vaezipour et al. 2022; Moretó‐Planas et al. 2023). However, evidence on the diagnostic performance of POCUS in children with presumptive TB remains limited. Few of these studies have assessed comprehensive POCUS (cPOCUS) incorporating multiple organ systems (lung, mediastinum, and abdomen) (Bigio et al. 2021), an approach of great interest given that, comparatively to adults, the most common findings in pediatric TB are mediastinal lymph nodes (Mahomed et al. 2023).

Understanding the potential role of POCUS in the pediatric TB diagnosis may inform its integration into pediatric TB diagnostic algorithms globally. Importantly, ultrasound is not expected to replace established pediatric TB diagnostic standards, which remain based on a composite of clinical assessment, microbiology, immunological testing, and conventional radiology. Rather, cPOCUS may serve as an adjunctive, radiation‐free tool to support detection of accessible intrathoracic or extrapulmonary abnormalities, particularly in settings where advanced imaging is limited.

The objectives of this study were therefore to: (1) evaluate the diagnostic performance of cPOCUS for detecting intrathoracic and extrathoracic TB abnormalities in children; (2) assess agreement between hospital radiologist findings and expert interpretations; and (3) compare cPOCUS findings with other imaging modalities (CXR and CT) and evaluate its diagnostic performance against the final clinical and microbiological TB classification.

## 2 Methods

### 2.1 Study Design and Setting

This was a prospective, observational study conducted in four tertiary hospitals in Spain (Hospital Sant Joan de Déu, Hospital Vall d'Hebron in Barcelona; and Hospital General Universitario Gregorio Marañón, Hospital Universitario 12 de Octubre in Madrid) between May 2023 and June 2024.

### 2.2 Study Population

The study population consisted of children aged 0–18 years presenting to the pediatric services (outpatient clinics, emergency department, and inpatient wards) of the participating hospitals during the study recruitment period. These hospitals are tertiary referral centers that provide routine diagnostic evaluation and care for children with suspected TB.

Children were screened for eligibility during routine clinical care and approached for recruitment if they met criteria for presumptive TB, defined as the presence of one or more of the following: (i) clinical signs or symptoms suggestive of TB (including persistent cough, fever, weight loss or failure to thrive, night sweats, lymphadenopathy, or fatigue); (ii) a history of close contact with a person with bacteriologically confirmed TB; (iii) a positive tuberculin skin test (TST) or interferon‐gamma release assay (IGRA); and/or (iv) CXR findings suggestive of TB as interpreted by the managing clinician or radiologist. Children were excluded if they had received TB treatment or TB preventive therapy for more than 72 h prior to enrolment, or if written parental or guardian informed consent and, where applicable, adolescent informed assent (≥ 12 years) were not obtained.

To characterize the study population, baseline demographic and clinical data were collected, including age, sex, referral source (outpatient, emergency, or inpatient), history and nature of TB exposure, and prior TB or TB preventive treatment. Internal controls were recruited from the same clinical settings during the same study period and included children in whom active TB was excluded following routine clinical, microbiological, and radiological evaluation. These may have included children with latent TB infection (LTBI), other respiratory or nonrespiratory diagnoses, or children investigated for TB in whom no evidence of disease was found.

### 2.3 Clinical and Laboratory Evaluation

All participants underwent standardized clinical evaluation and TB investigations as per hospital protocol. Diagnostic procedures included:

- TST and/or IGRA: An induration of ≥ 5 mm is considered positive in children who are close contacts of a suspected or confirmed TB case, in children with clinical and/or radiological findings suggestive of TB, in immunosuppressed children, or in those with documented TST conversion. An induration of ≥ 10 mm is considered positive in all other children, including those screened due to epidemiological risk factors or during routine evaluation, irrespective of prior BCG vaccination status. A positive IGRA result is defined according to the manufacturer's criteria and indicates *Mycobacterium tuberculosis* infection; results are interpreted in conjunction with clinical, epidemiological, and radiological findings (Baquero‐Artigao et al. 2023).
- GeneXpert MTB/RIF Ultra (Cepheid, Sunnyvale, CA, USA) and mycobacterial culture from sputum, gastric aspirates, or other relevant specimens when indicated.
- Human Immunodeficiency Virus (HIV) testing using rapid diagnostic assays was conducted in selected cases based on clinical indication and hospital protocol.
- CXRs (posteroanterior and lateral) were performed for all children. Thoracic CT scans were obtained only when clinically indicated (such as inconclusive CXR findings, assessment of complications), using iodinated intravenous contrast in every case. For CT, sedation was administered in younger children if necessary, according to institutional protocols to minimize motion artifacts and ensure high‐quality imaging.

### 2.4 POCUS Protocol

All hospital radiologists participating in the study received structured training in cPOCUS before study initiation. A two‐day workshop was conducted by the expert radiologist leading the study (X.S.‐C.), covering theoretical principles, standardized scanning techniques, and interpretation of findings relevant to pediatric TB. A detailed standard operating procedure (SOP) was developed to guide image acquisition, documentation, and reporting, ensuring consistency and reproducibility of cPOCUS examinations in different centers throughout the study period (Supporting Information 1: study ultrasound SOP) (Munyangaju et al. 2025; Heller et al. 2012; Tung‐Chen et al. 2021; Soldati et al. 2020).

cPOCUS was performed by hospital radiologists using portable ultrasound equipment. The scanning protocol included evaluation of lungs and pleura, mediastinum and abdomen. TB‐relevant regions and lesions were systematically assessed, including pleural effusion, subpleural consolidations, mediastinal and hilar lymphadenopathy (aortopulmonary window, paratracheal, subcarinal, and prevascular areas), and abdominal lymph nodes at the hepatic hilum, para‐aortic and celiac regions, as well as hepatosplenomegaly, focal hepatic/splenic lesions, and ascites. Each examination lasted 15–20 min and was recorded in real time (Supporting Information 2: study ultrasound CRF). Hospital radiologists were blinded to microbiological and radiological results at the time of scanning.

Anonymized ultrasound images and 5–10 s video clips, including Doppler acquisitions when relevant, were later independently reviewed by two expert radiologists (X.S.‐C. and D.B.), who were fully blinded to all clinical, laboratory, and radiological data. In cases where interpretations differed, a final conclusion was reached by consensus.

### 2.5 Case Classification

For pediatric TB diagnosis, cases were categorized following international standards after all diagnostic procedures including POCUS were performed, as follows (Graham et al. 2015):

- Confirmed TB: bacteriologically confirmed by Xpert test and/or culture.
- Probable TB: clinically diagnosed based on clinical and/or radiological findings (excluding cPOCUS), together with immunodiagnostic results and epidemiologic history
- Non‐TB disease: alternative diagnosis or absence of evidence supporting TB disease

For the purposes of diagnostic accuracy analyses, confirmed and probable TB were grouped as “TB disease,” and non‐TB as “no disease.”

### 2.6 Statistical Analysis

All analyses were performed using Stata version 18.5 (StataCorp, College Station, TX, USA). Categorical variables were compared using Fisher's exact test. Diagnostic accuracy metrics for cPOCUS (performed by hospital radiologists and interpreted by expert readers) were calculated using the final TB case classification as the reference standard. This composite classification was established without inclusion of cPOCUS results and incorporated clinical assessment, conventional radiography, immunological testing, and microbiological findings. cPOCUS findings were not included in the case definition, ensuring that ultrasound performance was evaluated independently as an adjunct modality.

As CT imaging was performed only in a subset of participants, CT was not used as a universal reference standard; rather, cPOCUS findings were compared descriptively with CT findings in those who underwent CT only. CXR was analyzed separately as a comparator imaging modality, rather than as a reference standard, because CXR contributed to the clinical case definition used to classify patients. Agreement between CXR and cPOCUS was evaluated using descriptive statistics and Cohen's kappa (*κ*). The study used Landis and Koch (1977) interpretation of kappa: < 0.20 for slight, 0.21–0.40 fair, 0.41–0.60 moderate, 0.61–0.80 substantial and > 0.80 almost perfect agreement level. All POCUS results were fully blinded and were not used in the clinical decision‐making process. The role of POCUS in this study was therefore to assess its diagnostic performance and potential utility as an adjunct tool, not to determine patient diagnosis. Statistical significance was set at *p* < 0.05.

Given the low incidence of pediatric TB in Spain and the prospective workflow requirements of comprehensive ultrasound acquisition and blinded expert review, this study was designed as a pilot feasibility evaluation. The final sample size (*n* = 27) reflects time‐bound recruitment across four tertiary hospitals between May 2023 and June 2024 rather than a formal power‐based calculation.

### 2.7 Ethical Considerations

The study was approved by the ethics board of the participating institutions. This study was conducted in conformity with ethical principles of all involved institutions and according to the declaration of Helsinki (WMA 2025).

## 3 Results

### 3.1 Study Population and cPOCUS Completeness

Twenty‐seven children were included (median age 9.0 years; 63% male), of whom 12 (44.4%) were classified as TB disease and 15 (55.6%) as non‐TB. All participants underwent CXR and cPOCUS, while CT was performed in 14 (51.9%).

cPOCUS examination completeness varied substantially: only 22.2% (6 out of 27) of examinations were fully complete, with partial lung or mediastinal assessment being the most common limitation. This low proportion of complete multi‐compartment examinations reflects real‐world operational constraints and likely contributed to missed abnormalities, thereby underestimating the potential sensitivity of cPOCUS under optimal protocol adherence.

### 3.2 Overall Imaging Findings and Contribution of cPOCUS

Mediastinal lymphadenopathy was the dominant imaging finding across all modalities. CXR detected abnormalities in 48.1% of children, primarily hilar and paratracheal lymphadenopathy. CT, when performed, identified more extensive multi‐station nodal disease and airway involvement. cPOCUS was particularly effective in detecting lymph nodes in the aortopulmonary (AOP) window and small subpleural consolidations, findings that were less consistently reported on CXR (Table 1, Tables S2–S4).

**TABLE 1:** Summary of imaging findings by modality and TB classification.

Imaging modality	TB cases (*n* = 12)	Non‐TB cases (*n* = 15)	Dominant patterns/added value
Chest X‐ray (CXR)	Abnormal in 9/12 (75.0%)	Abnormal in 4/15 (26.7%)	Predominantly mediastinal (hilar/paratracheal) lymphadenopathy; parenchymal opacities. Most sensitive modality overall.
Chest CT^a^	Abnormal in 7/9 (77.8%)	Abnormal in 2/5 (40.0%)	Multi‐station mediastinal lymphadenopathy, airway compression, atelectasis. Most detailed anatomical characterization.
cPOCUS—radiologist	Abnormal in 7/12 (58.3%)	Abnormal in 2/15 (13.3%)	Detection of accessible mediastinal nodes (especially AOP window) and small subpleural consolidations. Higher specificity than CXR.
cPOCUS—expert^b^	Abnormal in 7/11 (63.6%)	Abnormal in 3/15 (20.0%)	Improved detection of subtle AOP window lymph nodes (> 1 cm) and small lung consolidations; pattern consistent with radiologist readings.
Most frequent finding across modalities	_—_	—	Mediastinal lymphadenopathy was the dominant abnormality across CXR, CT, and cPOCUS; CT identified the greatest number of nodal stations, while cPOCUS preferentially detected AOP window nodes.

^a^ CT performed in a subset of participants only.

^b^ One cPOCUS examination was non‐interpretable for expert review.

Assessment of cPOCUS completeness, based on images and clips received for expert review, showed substantial variability in the extent to which different anatomical compartments were evaluated. Only 6 of the 27 children (22.2%) had a fully complete cPOCUS exam covering the mediastinum, lungs, and abdomen. The majority (20/27; 74.1%) had incomplete examinations, most often due to partial or missing lung ultrasound, incomplete mediastinal assessment, or omission of abdominal scanning. Incomplete lung evaluation was the most common limitation, occurring in both TB and non‐TB cases, whereas missing mediastinal views occurred less frequently but mainly among TB cases. One child (3.7%) had no cPOCUS images and video clip saved (Table S1).

### 3.3 Comparison Between Imaging Modalities

Overlap of abnormalities detected by CXR, cPOCUS, and CT is shown in Table 2. cPOCUS identified a subset of abnormalities concordant with CT, particularly AOP window lymphadenopathy, but did not visualize deep hilar or retrotracheal nodes or airway compression. In several cases, cPOCUS detected subtle abnormalities in CT‐normal children, potentially reflecting early disease or limited specificity.

**TABLE 2:** Comparison between cPOCUS (radiologist and expert) and CT among children with interpretable CT (*n* = 14).

	Radiologist cPOCUS normal	Radiologist cPOCUS abnormal	Expert cPOCUS normal	Expert cPOCUS abnormal
CT normal (*n* = 5)	4	1	3	2
CT abnormal (*n* = 9)	4	5	4	5

### 3.4 Diagnostic Performance

Diagnostic performance varied across imaging modalities. Using the final clinical classification as the reference standard, CXR demonstrated a sensitivity of 75.0% (95% CI: 42.8–94.5) and specificity of 73.3% (95% CI: 44.9–92.2), making it the most sensitive modality among the three. Radiologist cPOCUS showed a sensitivity of 58.3% (95% CI: 27.7–84.8) and specificity of 86.7% (95% CI: 59.5–98.3), while expert cPOCUS achieved a sensitivity of 63.6% (95% CI: 30.8–89.1) and specificity of 80.0% (95% CI: 51.9–95.7). Although associations were in the expected direction, confidence intervals were wide and estimates should be interpreted cautiously given limited power (Table 3).

**TABLE 3:** Diagnostic performance of imaging modalities with 95% confidence intervals^a^.

Test	Sensitivity % (95% CI)	Specificity % (95% CI)	PPV % (95% CI)	NPV % (95% CI)	Fisher's exact *p*
CXR abnormal	75.0% (42.8–94.5)	73.3% (44.9–92.2)	69.2% (39.0–90.9)	78.6% (49.2–95.3)	0.0213
cPOCUS abnormal (radiologist)	58.3% (27.7–84.8)	86.7% (59.5–98.3)	77.8% (40.0–97.2)	72.2% (46.5–90.3)	0.0369
cPOCUS abnormal (expert)^b^	63.6% (30.8–89.1)	80.0% (51.9–95.7)	70.0% (34.8–93.3)	75.0% (47.6–92.7)	0.0426

^a^ Reference standard: TB disease (Confirmed + Unconfirmed TB) versus Non‐TB (*n* = 27; expert cPOCUS *n* = 26).

^b^ Expert cPOCUS *n* = 26 due to one non‐interpretable scan.

### 3.5 Agreement Between Readers and Modalities

Agreement between CXR and cPOCUS was fair for radiologist interpretations and moderate for expert interpretations. Notably, agreement between radiologist and expert cPOCUS readings was exceptionally high (*κ* = 0.92), indicating near‐perfect concordance and consistent identification of mediastinal and pulmonary abnormalities between readers. Differences between readers were limited to a small number of cases involving subtle AOP window lymph nodes or minimal lung consolidations, suggesting that additional expertise increases detection of small but clinically relevant findings (Table 4, Table S5).

**TABLE 4:** Agreement between imaging modalities.

Comparison	*κ* (kappa)	Interpretation	Percent agreement (%)^a^	*n*
CXR vs. cPOCUS (radiologist)	0.40	Fair	70.4	27
CXR vs. cPOCUS (expert)	0.53	Moderate	76.9	26
Radiologist cPOCUS vs. Expert cPOCUS	0.92	Almost perfect	96.2	26

^a^ Cohen's *κ* with Landis and Koch interpretation; percent agreement calculated over interpretable examinations.

## 4 Discussion

In this pilot prospective evaluation, cPOCUS demonstrated modest sensitivity and relatively high specificity for selected TB‐related abnormalities when compared against a composite clinical, microbiological, and imaging reference standard. Given the small sample size, incomplete examinations, and wide confidence intervals, diagnostic accuracy estimates should be interpreted cautiously as exploratory and hypothesis‐generating rather than definitive. Interpretation by expert readers resulted in small improvements in sensitivity and specificity, suggesting that operator experience and familiarity with TB‐related sonographic findings may influence performance; however, these gains were incremental rather than substantial. Taken together, these exploratory findings indicate that cPOCUS may identify selected TB‐related abnormalities in children, but its diagnostic contribution was limited and non‐definitive.

A key observation is that cPOCUS reliably identified mediastinal lymphadenopathy, especially enlarged AOP window nodes, which emerged as the dominant abnormality across modalities. This aligns with existing pediatric TB literature from low and high‐burden settings, where ultrasound has proven useful in detecting intrathoracic and extrathoracic lymphadenopathy, pericardial effusion, and abdominal TB manifestations (Moretó‐Planas et al. 2023; Heuvelings et al. 2019; Bélard et al. 2018; Heller et al. 2017; Fentress et al. 2020; Fentress et al. 2022; Vonasek et al. 2024; Erem et al. 2023; Buonsenso et al. 2018; Buonsenso et al. 2019; Morello et al. 2022). In our cohort, cPOCUS also captured small focal pulmonary consolidations that were not always visible on CXR. However, cPOCUS missed abnormalities in four of the nine CT‐abnormal cases for both radiologist and expert readers, highlighting an important limitation: ultrasound cannot visualize deeper hilar, retrotracheal, or subcarinal lymph nodes or airway compression, readily identified by CT. These findings reinforce that cPOCUS adds value as a complementary imaging tool but cannot replace CT for evaluating deep mediastinal structures. Given the limited number of participants who underwent CT, these comparisons should be interpreted cautiously and as descriptive trends rather than formal accuracy estimates.

The diagnostic profile observed in this study, moderate sensitivity but relatively high specificity, likely reflects both anatomical and technical characteristics of ultrasound in pediatric TB. Ultrasound is inherently limited in its ability to visualize deep mediastinal and hilar structures, including retrotracheal or subcarinal lymph nodes, which are frequently involved in pediatric TB and are more readily detected by CT (Pool et al. 2017). This limitation may contribute to missed abnormalities and therefore lower sensitivity. In contrast, when cPOCUS identifies accessible findings such as enlarged aortopulmonary (Ao‐P) window lymph nodes or small subpleural consolidations, these abnormalities appear to be relatively specific for TB‐related pathology in the appropriate clinical context. Therefore, while cPOCUS cannot replace conventional imaging modalities, its high specificity for selected lesions suggests potential value as an adjunctive tool to support TB evaluation, particularly in settings where CT is unavailable or impractical.

The study demonstrated high agreement between radiologist and expert cPOCUS interpretations (*κ* = 0.92), suggesting good reproducibility of cPOCUS interpretation when the protocol is followed, although this finding should be interpreted cautiously given the small sample size. Differences between readers were minimal and largely confined to subtle AOP window nodes or small subpleural consolidations, which were more frequently detected by experts. Agreement between cPOCUS and CXR was fair to moderate, reflecting perhaps differences in the anatomical windows accessible to each technique.

An important operational insight emerged from the assessment of cPOCUS completeness. Despite nearly universal implementation, only 22.2% of examinations achieved full multi‐compartment coverage (mediastinum, lungs, and abdomen). Most examinations were incomplete often due to omitted lung regions, incomplete mediastinal views, or missing abdominal sweeps, reflecting real‐world workflow constraints. Several radiologists reported difficulty adhering to the full SOP, describing the protocol as lengthy and challenging to integrate into routine clinical schedules. This variability likely contributed to missed abnormalities and may have underestimated the full diagnostic potential of cPOCUS in this study. These findings highlight that protocol simplification, targeted training, and dedicated time for comprehensive scanning are essential for reliable implementation.

As with all ultrasound‐based techniques, cPOCUS remains inherently operator‐dependent. Diagnostic yield is influenced not only by interpretation expertise but also by image acquisition quality, protocol adherence, and the ability to consistently evaluate predefined anatomical windows. Although inter‐reader agreement was high in this study, generalizability to less specialized settings may require simplified protocols, tiered training, and robust quality assurance mechanisms.

From a health systems perspective, these observations emphasize that cPOCUS is not merely a technical innovation but a workflow‐sensitive diagnostic strategy. Its success and clinical utility depend on feasibility, sustained training in routine practice, standardized protocols, and quality assurance mechanisms to ensure reproducibility across different clinical settings and operator skill levels. A tiered training approach, starting with simplified, high‐yield scanning regions and gradually expanding to comprehensive protocols, may improve sustainability and diagnostic consistency.

This study has several limitations. The combination of small sample size and incomplete scan acquisition limits statistical power and may bias sensitivity estimates downward, restricting the ability to draw firm conclusions regarding diagnostic performance. Completeness of cPOCUS examinations was low, with many scans lacking full assessment of all predefined anatomical compartments, which likely biased diagnostic performance estimates and may have reduced the apparent sensitivity of cPOCUS. CT imaging was available for only half of the participants, restricting cross‐modality comparisons. Each participating hospital contributed only one primary reader, precluding a full assessment of intra‐observer variability. Variable adherence to the standard operating protocol may have further influenced cPOCUS performance, and the tertiary‐care setting in Spain may not reflect resource‐constrained contexts where POCUS could play a more central diagnostic role.

## 5 Conclusion

Across all imaging modalities, mediastinal lymphadenopathy emerged as the principal radiological hallmark of pediatric TB. While CT provided the greatest anatomical detail, cPOCUS offered complementary, portable, and radiation‐free diagnostic information particularly for detecting AOP window lymphadenopathy and small subpleural lesions. The high agreement between readers suggests that cPOCUS interpretation can be reproducible when standardized protocols are applied; however, performance remains dependent on operator expertise and examination completeness. cPOCUS may represent a promising adjunctive imaging tool for detecting accessible mediastinal lymphadenopathy and small peripheral lung lesions in children with presumptive TB. However, these preliminary findings require confirmation in larger, adequately powered studies with improved protocol adherence before routine diagnostic integration can be recommended.

## Author Contributions

Q.B., E.L.‐V., I.M., and I.T.‐C. contributed to study conceptualization and protocol development. A.N.‐J., A.S.‐G., A.S.‐A., J.M.E.F., M.E., B.S.G., A.H.‐L., A.M.L.Z., E.L., A.M., D.B., and E.A.P. were responsible for data collection. D.B. and X.S.‐C. served as expert reviewers. I.M. carried out data analysis and manuscript writing. All authors participated in manuscript revision and approved the final version of the manuscript.

## Funding

I.M., Q.B., E.L.‐V., and I.T.‐C. acknowledge the support from the grant CEX2018‐000806‐S funded by MCIN/AEI (Ministerio de Ciencia e Innovación)/10.13039/501100011033. I.M. acknowledges the support of a fellowship from the ‘La Caixa’ Foundation (ID 100010434). The fellowship code is LCF/BQ/DI21/11860045. E.L.‐V. acknowledges the support from European Respiratory Society and the European Union's H2020 research and innovation programme under the Marie Sklodowska‐Curie grant agreement No. 847462 and Beca de Investigación INVEST‐AEP 2022. Grants to A.N.‐J.: Carlos III Institute of Health, Ministry of Economy and Competitiveness of Spain (grant no. PI22/00766); and Spanish Society of Pneumology and Thoracic Surgery (grant no. 169‐2022). None of the funding sources had a role in the study's design, conduct and reporting. All other authors received no specific funding for this work. There was no additional external funding received for this study.

## Ethics Statement

The study was approved by the ethics board of the participating institutions: Hospital General Universitario Gregorio Marañón (24/04/2023, Acta 08/2023); CEIm Fundació Sant Joan de Déu (22/06/2023, acta 13/2023 reference: PIC‐109‐23); Hospital Universitario 12 de Octubre (28/02/2023, reference: 23/113); and Hospital Universitario Vall d'Hebron (28/04/2023, reference: PR(AMI)179/2023). This study was conducted in conformity with the ethical principles of all involved institutions and according to the declaration of Helsinki.

## Conflicts of Interest

The authors declare no conflicts of interest.

## Supporting information


**Data S1:** jcu70228‐sup‐0001‐Supinfo1.docx.

**Table S1:** Completeness of cPOCUS exams by TB category (*n* = 27).
**Table S2:** Summary of main findings across all imaging modalities.
**Table S3:** Imaging findings among children with TB disease only (*n* = 12).
**Table S4:** Side‐by‐side comparison of TB vs non‐TB cases.
**Table S5:** Comparison of radiologist vs expert cPOCUS findings by anatomical compartment.

## Data Availability

The data that support the findings of this study are available from the corresponding author upon reasonable request.
