# Supplementary material for: Diagnostic Performance of Comprehensive Point‐of‐Care Ultrasound for Pediatric Tuberculosis in Spain: A Prospective Observational Study
Source: J Clin Ultrasound. 2026 Mar 15;54(6):1425–32. doi: 10.1002/jcu.70228 (PMC13332548; doi:10.1002/jcu.70228)
Supplement: Supplementary file 1 — Data S1: jcu70228‐sup‐0001‐Supinfo1.docx. [file JCU-54-1425-s001.docx]

# **IMAGINE study ultrasound SOP**

# TABLE OF CONTENTS:

Table of Contents

[Study Title: IMAGINE 1](#_Toc134022028)

[TABLE OF CONTENTS: 2](#_Toc134022029)

[1. Introduction 4](#_Toc134022030)

[2. Prerequisites 4](#_Toc134022031)

[3. Preparation 4](#_Toc134022032)

[4. Equipment 4](#_Toc134022033)

[4.1. Scan equipment 4](#_Toc134022034)

[4.2. Scan Accessories 5](#_Toc134022035)

[5. Lung ultrasound 5](#_Toc134022036)

[5.1 Sonographer and patient positioning 6](#_Toc134022037)

[5.2 Regions covered 6](#_Toc134022038)

[5.3 General machine settings and probe selection 7](#_Toc134022039)

[5.4 General approach to scanning 7](#_Toc134022040)

[5.5 Specific approach per region to lung scanning 8](#_Toc134022041)

[5.6 Definitions of scan findings 9](#_Toc134022042)

[5.7 Image acquisition and tagging 10](#_Toc134022043)

[6. Abdominal Ultrasound 11](#_Toc134022044)

[6.1 Sonographer and patient positioning 11](#_Toc134022045)

[6.2 Patient preparation 12](#_Toc134022046)

[6.3. Examination 12](#_Toc134022047)

[6.5 Definitions of scan findings 20](#_Toc134022048)

[6.6 Image acquisition 21](#_Toc134022049)

[7. Mediastinal lymph node ultrasound 21](#_Toc134022050)

[7.1 Sonographer and Patient positioning 21](#_Toc134022051)

[7.2 Regions covered 22](#_Toc134022052)

[7.4 Definition of scan findings 23](#_Toc134022053)

[7.6 General approach and specific approach 23](#_Toc134022054)

[Definitions of scan findings 27](#_Toc134022055)

[Image acquisition 27](#_Toc134022056)

[8. Assessing scan quality and other general assessments 28](#_Toc134022057)

[8.1 Quality 28](#_Toc134022058)

[8.2. Other 28](#_Toc134022059)

[9. Completing the US exams and documenting the findings 28](#_Toc134022060)

# Introduction

**Scope**:

This Standard Operating Procedure (SOP) is for use by pediatric radiology specialists when performing the:

A. lung,

B. focused abdominal and

C. mediastinal ultrasound on study participants.

**Purpose:**

Lung ultrasound (US) is sensitive and specific for the diagnosis of pneumonia in children. However, little has been published on the use of mediastinal, lung US and focused abdominal US in patients with pulmonary tuberculosis (PTB). US will be used in this study to evaluate the lung, pleural cavity, abdomen and mediastinum for abnormalities that may be related to PTB or extra-pulmonary TB (EPTB).

# Prerequisites

Prior to beginning the US exam, the nature of the exam must be explained to the caregiver of the child (and to the child participant if age appropriate). All accessories required to perform the US should be available. All steps must be taken to ensure the child participant’s privacy, including the exam being conducted in a private room. Infection prevention and control measures should also be considered in the context of patients with suspected or known tuberculosis.

# Preparation

- Explain the nature of the exam to the child’s caregiver, and to the child if age appropriate.
- Ensure informed consent has been obtained.
- Participant information should be entered into the US machine so that the US study data will display the participant ID (PID) number, date and time.
- Open a new US Case Report Form (eCRF) on the tablet and label with the PID, date and time in preparation for data capture. If electronic capture is not possible then enter these details on a paper version of the CRF.

# Equipment

## 4.1. Scan equipment

| 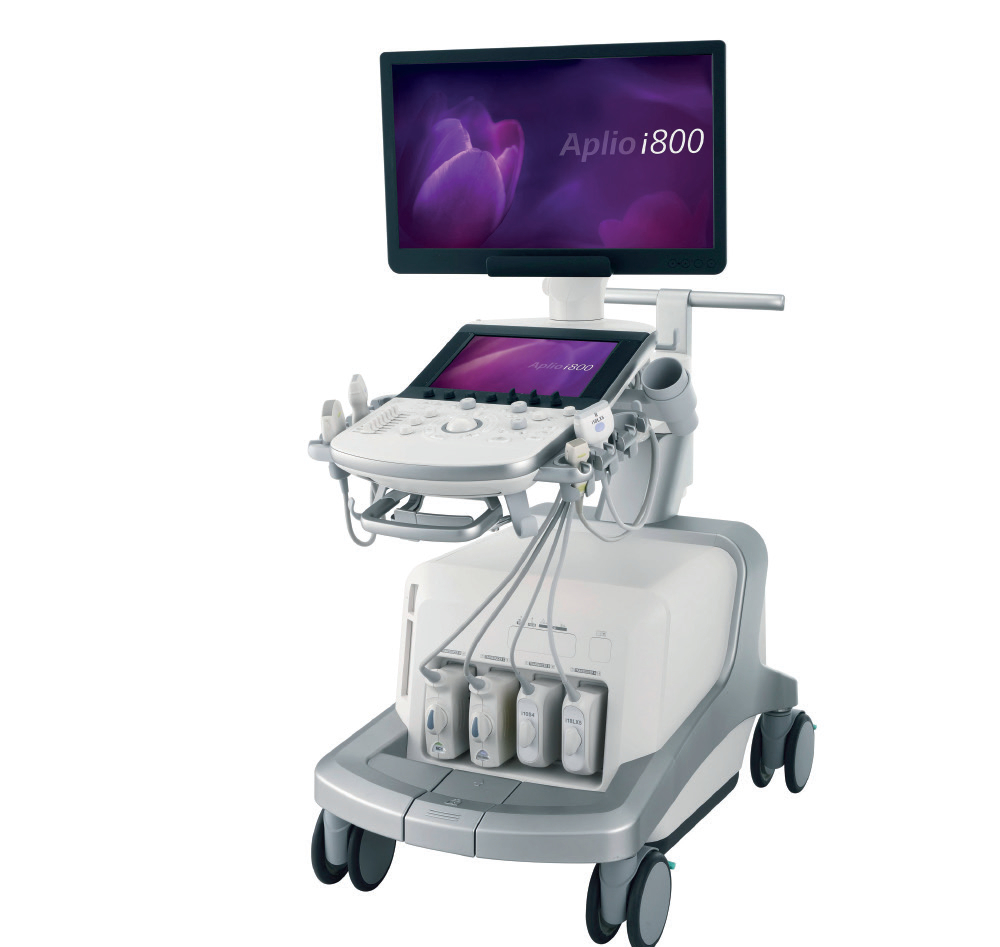 | **A fixed scan in the radiology department (similar to the picture)** |
| --- | --- |
| 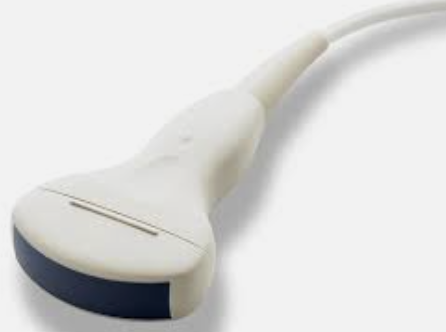 | **CONVEX PROBE:**  Ideal for abdominal scan, also possible for lung scan in older children. |
| 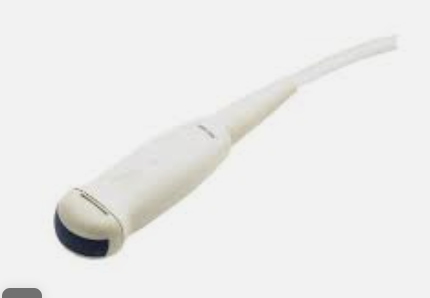 | **MICROCONVEX PROBE:**  You can perform all the exams of this SOP with this probe. |
| 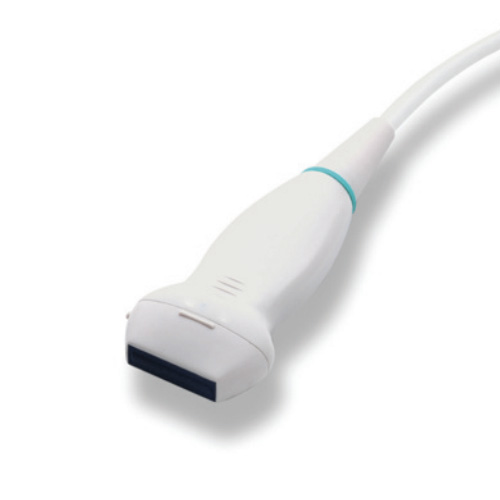 | **LINEAR PROBE:**  Can be use for children for mediastinal exam |

## 4.2. Scan Accessories

- Ultrasound gel
- Exam bed
- Paper towel / tissue
- Disinfectant / germicide wipes
- Masks as required
- eCRF and /or Paper CRFs for data capture

# Lung ultrasound

## 5.1 Sonographer and patient positioning

In most instances, the sonographer will be positioned to the right side of the participant.

Anterior and lateral lung views will ideally be acquired with the participant supine on the exam couch (alternatively, on the caregiver’s lap). Posterior images will ideally be acquired with the patient lying on his/her side on the examination couch (alternatively sitting on the bed or the caregiver’s lap). In young children ideal positioning may not be possible, and the child should be placed in the position that is most comfortable and that allows best access to the region of interest.

## 5.2 Regions covered

Scanning will be performed from the lung apices to the diaphragm, on each side. For the purposes of documentation, the chest will be divided into the 14 regions described below:

| **Abbrev.** | **Full Name** | **Description** |
| --- | --- | --- |
| R1 | Right Anterior Upper | Sternum to anterior axillary line, superior half |
| R2 | Right Anterior Lower | Sternum to anterior axillary line, lower half, includes diaphragm edge |
| R3 | Right Lateral Upper | Anterior axillary line to posterior axillary line -superior half |
| R4 | Right Lateral Lower | Anterior axillary line to posterior axillary line, lower half, includes diaphragm edge |
| R5 | Right Posterior Upper | Transverse process to posterior axillary line, superior third |
| R6 | Right Posterior Middle | Transverse process to posterior axillary line, middle third |
| R7 | Right Posterior Lower | Transverse process to posterior axillary line, lower third, includes diaphragm edge |
| L1-7 |  | As for right hemithorax |

**Protocol reference source:**

- Soldati G, Smargiassi A, Inchingolo R, Buonsenso D, Perrone T, Briganti DF, et al. Proposal for international standardization of the use of lung ultrasound for patients with covid-19: a simple, quantitative, reproducible method. J Ultrasound Med. 2020;39(7):1413–1419. <https://doi.org/10.1002/jum.15285>
- Tung-Chen Y, Ossaba-Vélez S, Acosta Velásquez KS, Parra-Gordo ML, Díez-Tascón A, Villén-Villegas T, Montero-Hernández E, Gutiérrez-Villanueva A, Trueba-Vicente Á, Arenas-Berenguer I, Martí de Gracia M. The Impact of Different Lung Ultrasound Protocols in the Assessment of Lung Lesions in COVID-19 Patients: Is There an Ideal Lung Ultrasound Protocol? J Ultrasound. 2022 Sep;25(3):483-491. doi: 10.1007/s40477-021-00610-x. Epub 2021 Dec 2. PMID: 34855187; PMCID: PMC8638638. <https://www.ncbi.nlm.nih.gov/pmc/articles/PMC8638638/>

## 5.3 General machine settings and probe selection

- Use convex or linear transducers, according to the patient's body size.
- Doppler ultrasound may be used to investigate areas of consolidation for potential breakdown / cavitation

## 5.4 General approach to scanning

- Probe position:
  - - The mark of the probe will be facing up for the longitudinal cuts and towards the sonographer in transversal cuts.
- Longitudinal cut will be considered any cut that follows the longitudinal axis of the patients.
- Transversal cut will be considered any cut that is perpendicular to the longitudinal axis.
- The chest scanning will be done per the 14 regions previously described.
- The chest scanning will start with the probe in the longitudinal axis to use the ribs as an anatomic reference.
- Then a free scanning of the region will take place before taking the pictures and clips
- Details relating to which clips and images will be recorded are outlined in Section 5.7

## 5.5 Specific approach per region to lung scanning

- - - Right anterior lung (regions R1-R2)

With the participant in supine, place the longitudinally orientated probe in a right parasternal position in the intercostal space immediately below the clavicle. Freely scan the region to look for abnormalities described in the ***5.6 section***.

Find a representative image of the region in the longitudinal position, freeze and tag the image and save a **1^st^ still picture**. Then rotate the probe into a transversal position with the mark pointing to you and take a representative picture of the region by freezing the image, tagging it and saving the **2^nd^ still picture**, preferably avoiding the ribs’ shadow. With the probe still in the longitudinal position, scan slowly, from medial to lateral, until reaching the anterior axillary line, then freeze the image, name it and save a **5 seconds clip**. Ideally the scan should include 3 or more intercostal spaces in the screen.

Sequentially move down the anterior chest wall into R2 region and repeat the process in similarly fashion.

By the end of this scan we should have at least 2 videos, one per region, and 4 pictures, but if anything, pathological is identified the sonographer should find the best image to represent the alteration. For consolidations it is advised to have one additional clip with color flow, where possible.

- - - Right lateral lung (regions R3-R4)

With the participant’s right arm in “L” position (90º position) from the shoulder’s high, place the longitudinally orientated probe at the level of the anterior axillary line, in the highest accessible intercostal space. Perform a free scan of R3 region, then look for abnormalities described in the 5.7 section. ***Repeat similar procedure as previously described for R1-R2 for acquisition of 2 still picture and 1 clip***.

- - - Right posterior lung (regions R5- R7)

With the participant in a left lateral decubitus position, and the right arm positioned to make sure the scapula is not on the way, place the longitudinally orientated probe in a right paraspinal position, in the intercostal space immediately below the tip of the scapula and perform a free scan of R5 looking for abnormalities. ***Repeat similar procedure as previously described for R1-R2 for acquisition of 2 still picture and 1 clip***.

- - - Left lung

Repeat technique described in sections for R1-R7 for the left lung.

## 5.6 Definitions of scan findings

- - - Normal lung pattern: pattern showing normal respiratory sliding and presence of A-lines without interstitial pattern, consolidation, air bronchogram or effusion. The pleural line should not be thickened or interrupted. A-lines are horizontal lines equally spaced from the pleural line. This is an artifact generated by subpleural air and is a normal finding. A-lines may not always be well visualized despite otherwise normal lung pattern. To note that although A-lines are not always clearly visualized, the lung pattern can still be described as normal if other criteria described here are met. Two or fewer B lines can be seen in one intercostal space
    - B-lines: vertical lines originating at the pleural line or from a sub-pleural consolidation (SPC). Document how many B-line are visualized per intercostal space, whether they are discreet or confluent, and document whether they originate from the pleural line or from a SPC. Three or more B lines, or confluent B lines are considered pathological. and understood to represent interstitial lung pattern.
    - Pleural line: the pleural line can be interpreted with the probe either in the transverse or the longitudinal position. Document if the pleural line is abnormal, and whether pleural gliding is absent, the pleura is thickened, there is presence of air (pneumothorax), fluid (pleural effusion) or other abnormalities.
    - Pleural effusion: an anechoic collection between the pleural line or diaphragm and the chest wall. If present, record whether the effusion is:
    - simple (anechoic collection without stranding, loculations or septations) or
    - complex (heterogenous with septations, loculations or fibrinous strands)

Measure the maximum depth of the effusion in each lung region as per the CRF.

- - - Subpleural consolidation: Hypoechoic or echo-poor rounded or rectangular subpleural focus, measuring less than 5 mm in maximum dimension, which abuts the pleura, interrupts the pleural line and may have an associated comet-tail artefact or B-line. Presence and number of SPC’s should be documented.
    - Consolidation: defined as a subpleural, echo-poor or tissue-like region ≥ 5 mm in maximum dimension, with or without sonographic air bronchograms. If a consolidation is identified then it should be measured (length = largest surface extension along the pleura, by depth = largest depth from the pleura towards the bottom of the US screen), the number of consolidations should be recorded, and Doppler should be used to establish if there is associated breakdown or cavitation. If a consolidation overlaps into adjacent region, then measure only in the predominant region.
    - Air bronchogram: air-filled bronchi that become visible on ultrasound when the tissue density of surrounding alveoli increases due to pathology. They are identified by multiple hyperechoic densities surrounded by hypoechoic consolidated lung parenchyma.
    - Fluid bronchogram: represent the presence of fluid attenuation material within respiratory bronchioles with surrounding collapsed or consolidated lung. On ultrasound they are seen as anechoic tubular structures representing fluid-clogged bronchi within relatively echogenic collapsed lung. The absence of Doppler flow confirms that these are not vessels but airways.

## 5.7 Image acquisition and tagging

- Standard image acquisition

**At least one representative longitudinal still image, one transversal still image and a 5-10-second video clip must be routinely recorded for each lung region**. Therefore, a ***total of 28 still images (14 longitudinal and 14 transverse) and 14 video clips*** should be routinely obtained for each lung US exam.

**Each clip should be labeled according to the table below**:

| **Abbrev.** | **Full Name** | **LABELS** |
| --- | --- | --- |
| R1 | Right Anterior Upper | **R1 L**: Longitudinal image on R1  **R1 T**: Transversal image on R1  **R1**: The clip with the probe in a longitudinal position from medial to lateral |
| R2 | Right Anterior Lower | **R2 L**: Longitudinal image on R2  **R2 T**: Transversal image on R2  **R2**: The clip with the probe in a longitudinal position from medial to lateral |
| R3 | Right Lateral Upper | **R3 L**: Longitudinal image on R3  **R3 T**: Transversal image on R3  **R3:** The clip with the probe in a longitudinal position anterior axillary line to posterior axillary line |
| R4 | Right Lateral Lower | **R4 L**: Longitudinal image on R4  **R4 T**: Transversal image on R4  **R4**: The clip with the probe in a longitudinal position anterior axillary line to posterior axillary line |
| R5 | Right Posterior Upper | **R5 L**: Longitudinal image on R5  **R5 T**: Transversal image on R5  **R5**: The clip with the probe in a longitudinal position para spinal to posterior axillary line (medial to lateral) |
| R6 | Right Posterior Middle | **R6 L**: Longitudinal image on R6  **R6 T**: Transversal image on R6  **R6**: The clip with the probe in a longitudinal position para spinal to posterior axillary line (medial to lateral) |
| R7 | Right Posterior Lower | **R7 L**: Longitudinal image on R7  **R7 T**: Transversal image on R7  **R7**: The clip with the probe in a longitudinal position para spinal to posterior axillary line (medial to lateral) |
| L1-7 |  | As for right hemithorax |

- - - Extra image acquisition

If there are any abnormalities noted anywhere in a region, a clip should be saved of each of the pathologic findings. In these cases, there may be more than one clip recorded per region, and these should then be labelled as R1.1 and R1.2, for Region 1, and so forth. In addition to the clips, a still image should also be obtained of the pathological area, and all consolidations, SPC and cavities should be measured and annotated on this image.

# Abdominal Ultrasound

**Protocol reference source:**

Heller T, Wallrauch C, Goblirsch S, Brunetti E. Focused assessment with sonography for HIV-associated tuberculosis (FASH): a short protocol and a pictorial review. Crit Ultrasound J. 2012 Nov 21;4(1):21. doi: 10.1186/2036-7902-4-21. <https://doi.org/10.1186/2036-7902-4-21>

## 6.1 Sonographer and patient positioning

The study will ideally be performed with the sonographer to the right of the participant

## 6.2 Patient preparation

Patient is placed in a comfortable supine position on the exam bed (or on the caregiver’s lap). A curved 3.5 to 5-MHz transducer is used for the examination of the abdomen. Sufficient amount of ultrasound gel is placed on the transducer and the patient’s abdomen to ensure good acoustic coupling between the probe and the skin. For the beginning of the examination (probe position 1a and 1b), the patient is asked to place his/her arms beside the body to relax the muscles of the abdominal wall.

## 6.3. Examination

The patient is scanned by six different regions (as shown in figure below). In each position, different questions are addressed:


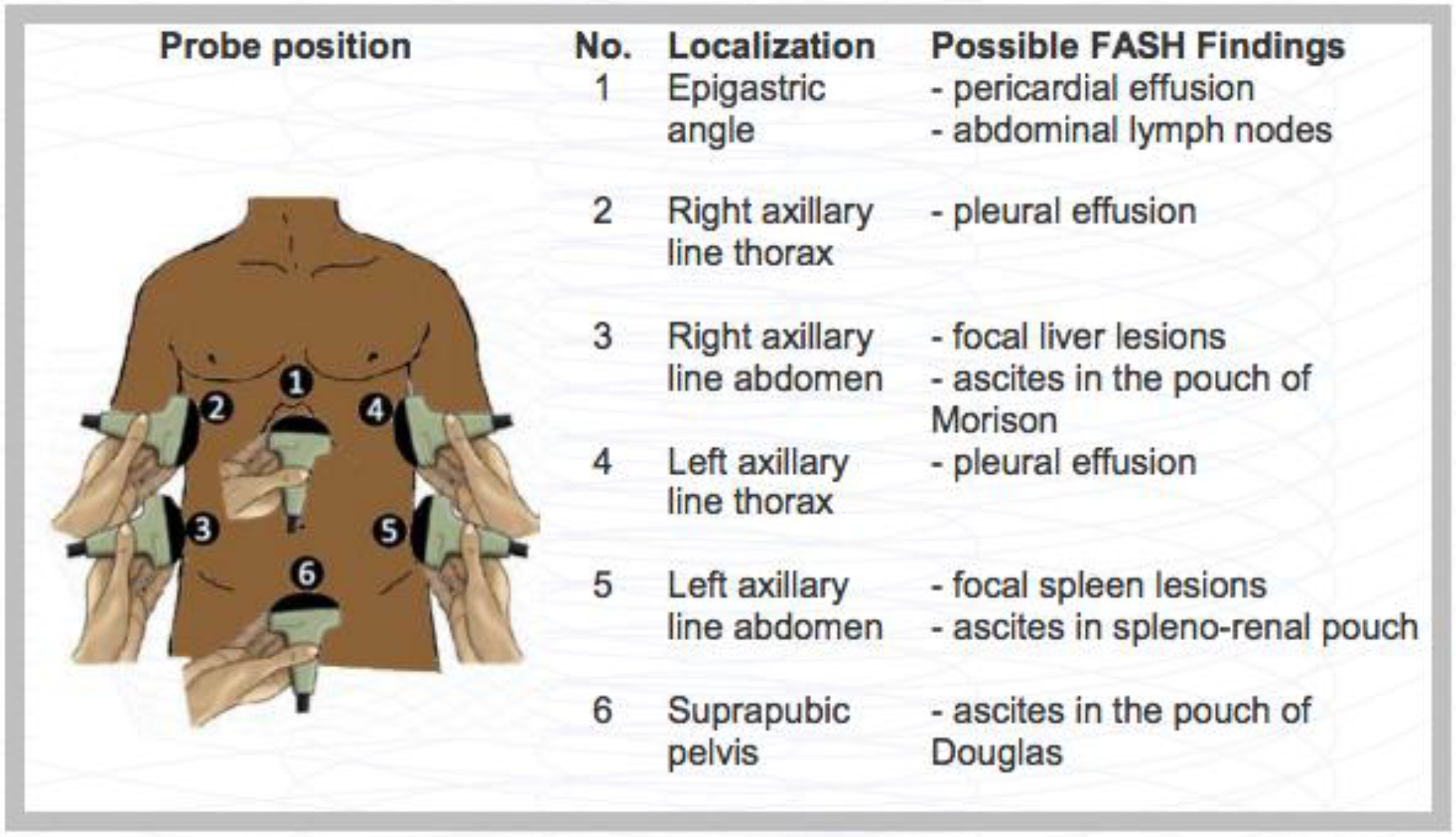


**Probe position 1a: pericardial effusion**

1. **Indication**: Pericardial effusion should be considered in every HIV-positive individual with signs of breathlessness, retrosternal pain, and signs of progressive heart failure, tachycardia or hypotension. A chest X-ray showing cardiomegaly is frequently caused by pericardial effusion and should be further assessed by ultrasound.
2. **Probe position**: The probe is placed transverse in the epigastric angle; the transducer is then tilted cranially to get a view of the intra-thoracic organs and the heart. The patient should try to relax his abdominal muscles (arms placed beside the body) as an attempt is made to ‘scoop’ the transducer under the ribs. Commands asking the patient to inspire might help to displace the heart caudally and improve visualization. ***A still image of the intrathoracic organs and heart should be obtained in this transverse view and a 5 second video clip***.
3. **Normal findings**: The left lobe of the liver will be visible in the upper parts of the image serving as an acoustic window. Below this, the right atrium as well as the right ventricle will be seen and can be easily recognized by the rhythmic contractions. The right ventricle increases in size during diastole and contracts during the systolic phase of the heart action. The pericardium is seen as an echogenic structure between the liver and the heart; the parietal and visceral pericardium are inseparable (***Figure 2a***). In the distant parts of the US image, the left heart may be seen.


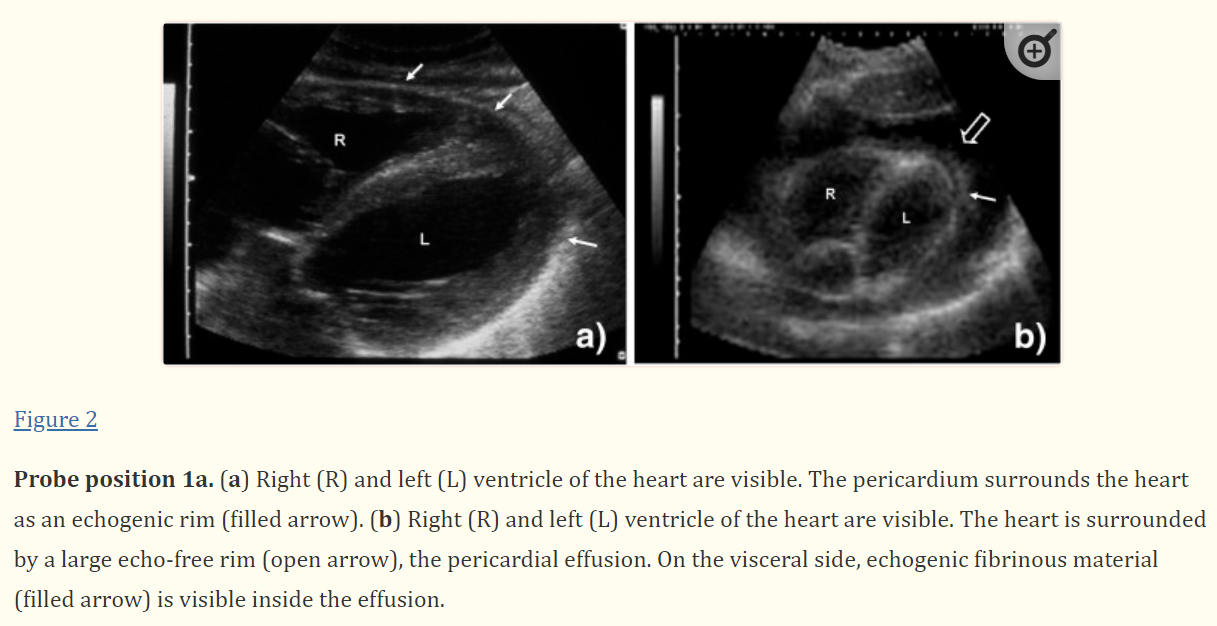


1. **Pathological findings**: Pericardial effusion mainly shows as an anechoic, black rim around the heart separating the visceral and parietal pericardia ​(***Figure2b***). The rim might surround the entire heart and be visible on the side of the left ventricle as well. Frequently, echogenic material like fibrin streaks can be seen floating in the anechoic effusion; occasionally, the whole effusion appears echogenic mainly due to high protein and particle content in purulent exudative effusions. In cases of pericardial tamponade, tachycardia and impaired filling of the right atrium and/or ventricle might be seen. Sonographic changes of tamponade, mainly diastolic collapse of the right ventricle due to increased pericardial pressure, might in some cases precede clinical symptoms and would then be termed ‘pre-tamponade’.
2. **Interpretation**: An anechoic rim surrounding the heart confirms the presence of a pericardial effusion. In a setting where TB has a high prevalence like in Sub-Saharan Africa, pericardial TB is the most frequent cause of pericardial effusion. Other differential diagnoses such as malignancies, e.g., lymphoma or Kaposi's sarcoma (KS) should be considered, especially in patients with suggestive lesions in other parts of the body. A thorough examination of the skin, the intra-oral mucosa, and lymph nodes should be done.

**Probe position 1b: abdominal lymph nodes**

1. **Indication:** Disseminated abdominal TB should be considered in all HIV-positive patients with fever, unexplained loss of weight, weakness, diarrhea, and/or abdominal pain. Patients occasionally complain of longstanding hiccups. Abdominal masses and ascites may be found during clinical examination. The patients often show advanced immunosuppression with very low CD4 counts.
2. **Probe position**: The transducer is tilted back to a position more or less perpendicular to the patient's skin. In this position, the upper abdominal/periportal area can be visualized. The transducer is then slowly moved caudally to assess the periaortic area. To minimize the distance between the probe and the retro-abdominal areas assessed, the abdominal wall should still be relaxed and the arms placed beside the abdomen. ***A still image of the upper abdominal/periportal area*** ***and a still image of the periaortic area should be obtained in this view and a 5 second video clip***.
3. **Normal findings**: Many physiological structures can be seen which are beyond the scope of the FASH examination. The examiner should attempt to locate the abdominal aorta as a landmark structure (***Figure 3a***). Once found, this structure should be followed caudally for approximately 10 to 15 cm.


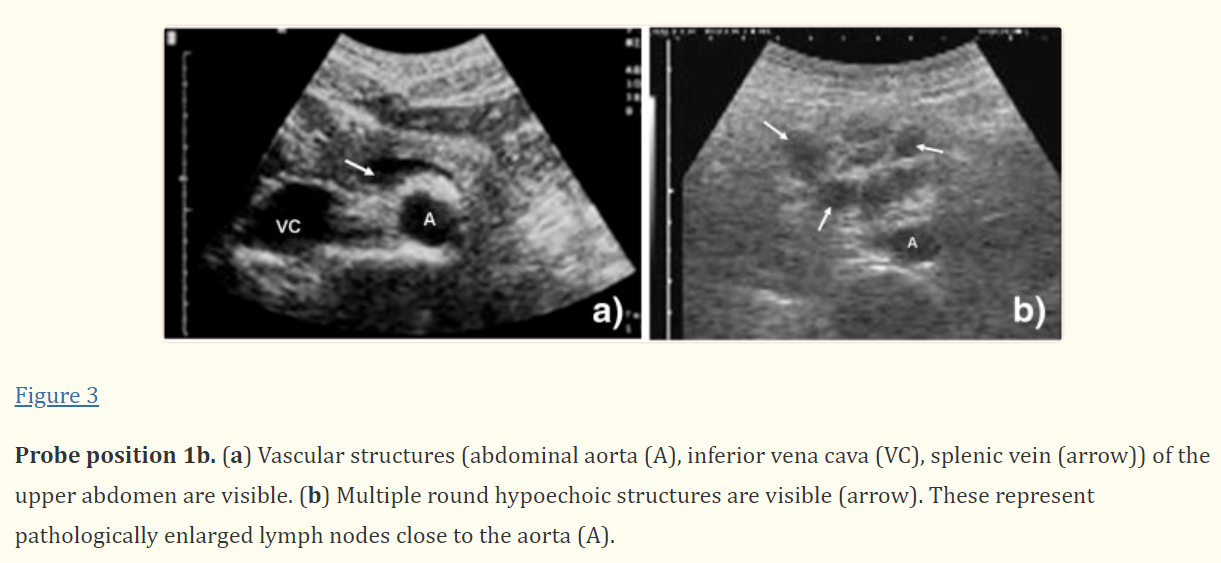


1. **Pathological findings**: Lymph nodes larger than 1.5 to 2 cm could be considered pathologic in an HIV-infected individual. Lymph nodes are usually hypoechoic (dark) round structures which appear in the US image (***Figure3b***), increase in size as the transducer moves towards the center of the nodes, and then grow smaller again as the opposite site is reached. This phenomenon of ‘growing’ and ‘shrinking’ as the probe passes the node is easily recognized and gives the lymph nodes a blinking appearance. The node is measured at its maximal dimension.
2. **Interpretation**: Enlarged abdominal lymph nodes in a HIV-positive African patient with low CD4 counts are suggestive of abdominal TB. A chest X-ray should be done as the majority of patients with abdominal TB also have pulmonary changes suggestive of the disease.

**Probe position 2: pleural effusion right side**

1. **Indication**: Pleural effusion should be considered in an HIV patient with pleuritic pain, irritating cough, or breathlessness. Dullness to percussion and reduced sounds of air entry are suggestive clinical findings; a chest X-ray might show opacity and displacement of the mediastinum to the contralateral side. Pleuritic TB may also be seen in patients with milder immune-suppression and even normal CD4 counts.
2. **Probe position**: The patient is asked to put his arms behind the head to free access to the side of the body. Additionally, this position widens the space between individual ribs. The transducer is positioned dorsal of the right mid-axillary line at the caudal part of the thorax. The transducer has to be adjusted so that the long axis is parallel to the ribs, allowing a trans-costal view. It is important to place the transducer as dorsal as possible (close to the examination couch), as fluid collects in the dependent parts due to gravity. ***A still image of the trans-costal view should be obtained and a 5 second video clip***.
3. **Normal findings**: The diaphragm and apical parts of the liver should be visible. If no effusion is present, the air in the basal parts of the lung will cause artifacts resembling a curtain that moves up and down with the respiratory cycle.
4. **Pathological findings**: Anechoic, black fluid may be visible in the costophrenic angle (***Figure 4b***). This is a pleural effusion, which is normally completely echo free but may contain internal echoes such as strands or smoke which are due to fibrinous structures or cells within the effusion.


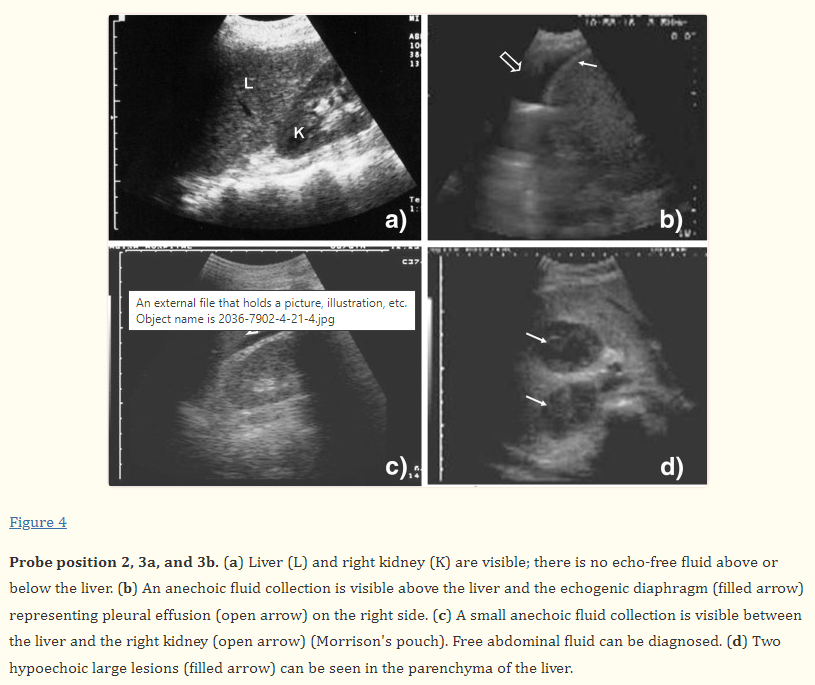


1. **Interpretation:** A pleural effusion in HIV-positive patients is very suggestive of TB, especially when unilateral. Possible differential diagnoses of pleural effusions, particularly in bilateral effusions are generalized KS and other malignancies. KS often produces bloody or serosanguinous fluid when aspirated upon thoracentesis. Parapneumonic effusions should be included in the differential diagnosis. Patients usually have a high fever, show infiltrates on chest-X-ray along with a leukocytosis. Congestive heart failure needs to be considered as a reason for pleural effusion which would be supported by the sonographic demonstration of reduced left ventricular contraction.

**Probe 3a: ascites in the hepato-renal pouch (Morrison’s pouch)**

1. **Indication**: see probe position 1b
2. **Probe position**: The transducer is moved a few centimeters caudally and is slightly rotated so that the longitudinal axis is parallel to the long axis of the body. Again, it is important to bring the scanner dorsally to see fluid in the dependent parts of the abdominal cavity. ***A still image of the pouch should be obtained and a 5 second video clip***.
3. **Normal finding**: The caudal edge of the liver and the right kidney should be visible. Between the two organs, there is usually an echogenic, white line (***Figure ​4a***).
4. **Pathological findings**: If echo-free, black fluid is visible between the liver and the kidney (***Figure ​4c***) or around the kidney. This is due to the free fluid in the abdominal cavity, i.e. ascites. Sometimes echogenic material like streaks or strands can be seen floating in the fluid, which might represent fibrin.
5. **Interpretation:** Ascites can be due to a number of reasons. In the HIV-infected patient, abdominal TB is one of the reasons, but other causes such as liver cirrhosis due to chronic hepatitis virus infection need to be considered. The finding of ascites needs to be interpreted in light of other clinical and laboratory findings. If possible, providers should attempt to assess sonographic signs of liver cirrhosis like a nodular surface, decreased vascular markings, and splenomegaly due to portal hypertension. These findings might be difficult to interpret for the less-experienced examiner.

**Probe position 3b: focal liver lesions**

1. **Indication**: see probe position 1b
2. **Probe position**: The transducer is moved slightly up again to be positioned in the area of the liver. Again, the axis of the transducer needs to be adjusted to be parallel to the ribs to avoid artifacts from the bony structures of the thoracic cage. ***A still image of the liver view should be obtained and a 5 second video clip***.
3. **Normal finding**: The homogenous tissue of the liver is visible; inside, the hepatic vessels can be seen. The details of the liver anatomy lie beyond the FASH examination.
4. **Pathological findings**: Focal hypoechoic lesions might be seen and are relatively easy to recognize if they are large (***Figure ​4d***).
5. **Interpretation**: In the tropical setting, hepatic abscesses are a common cause of abdominal complaints, especially in immunocompromised patients. These might be due to TB; however, other causes, especially amebic and bacterial abscesses, need to be considered, and in particular amebic abscesses are seen frequently.

**Probe position 4: pleural effusion left side**

1. **Indication**: see probe position 2.
2. **Probe position**: Mirroring the probe position 2, the transducer is placed on the left side of the thorax to assess for left pleural effusions. ***A still image of this view should be obtained and a 5 second video clip.***
3. **Normal finding**: The spleen and the left diaphragm should be visible; otherwise, the image is similar to 2.
4. **Pathological findings**: see probe position 2 (***Figure ​5b***).


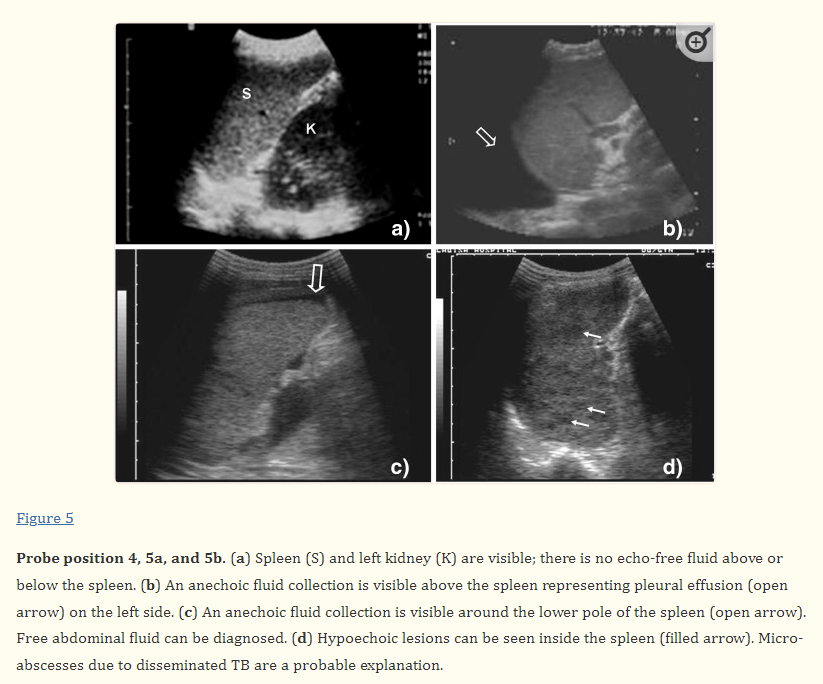


1. Interpretation: see probe position 2

Probe position 5a: ascites in the spleno-renal pouch

1. **Indication:** see probe position 1b.
2. **Probe position:** Mirroring the probe position 3a, the transducer is placed on the left flank to examine for free abdominal fluid in the dependent parts of the left abdominal cavity, i.e., between the spleen and kidney, and around the kidney. To achieve this, the transducer is moved caudally, and the long axis of the transducer needs to be more or less parallel to the long axis of the patient's body. ***A still image of the spleno-renal pouch view should be obtained and a 5 second video clip***.
3. **Normal finding:** The spleen and the left kidney should be visible to ensure correct probe position (***Figure 5a***). Both organs should be separated by an echogenic, white line representing the capsules of the organs.
4. **Pathological findings**: Analogous to the hepato-renal pouch on the right side of the abdomen, free fluid might collect in the spleno-renal pouch and is visible as an anechoic, black fluid (***Figure ​5c***).

1. **Interpretation**: Free fluid in the spleno-renal pouch represents ascites. As mentioned above, other causes of ascites should to be considered beside abdominal TB (see probe position 3a).

**Probe position 5b: focal splenic lesions**

1. **Indication:** see probe position 1b.
2. **Probe position**: Mirroring the probe position 3b, the transducer is moved slightly upwards and turned to be parallel to the ribs using the intercostal space as a window. ***A still image of the spleen view should be obtained and a 5 second video clip***.
3. **Normal finding**: The homogenous tissue of the spleen should be visible.
4. **Pathological findings**: Hypoechoic, dark lesions in the spleen (size approximately 0.5 to 2 cm) (***Figure ​5d***).
5. **Interpretation**: Multiple hypoechoic lesions may represent small abscesses, which are frequently secondary to disseminated TB. The image is characteristic and other differential diagnoses like other infections or disseminated malignancy are less likely. As mentioned previously, diagnostic steps for concomitant pulmonary TB (sputum, chest X-ray) should be done.

**Probe position 6: ascites in the pouch of Douglas**

1. **Indication**: see probe position 1b.
2. **Probe position**: The probe is placed on the lower abdomen touching the upper rim of the symphysis pubis. The pelvic region can be scanned in the longitudinal axis (long axis of the scanner parallel to the long axis of the patient) or in the transverse axis (long axis of the scanner parallel to the upper rim of the pelvic bone). For visualization of parts deeper in the pelvis, the transducer has to be tilted upwards so that the caudal structures of the small pelvis become visible. ***Still images (2) of the longitudinal axis view and a still image of transverse axis should be obtained and a 5 second video clip***.
3. **Normal finding**: The bladder should be visible and its size will vary depending on how full it is. As urine is a fluid, it will be visible as an echo-free, black area, and care has to be taken not to confuse this with free fluid. Behind the bladder, the uterus might be visible as a pear-shaped organ in the female patient. Behind these structures and in front of the rectum, which cannot be seen due to air artifacts, the pouch of Douglas is located (Figure 6a).


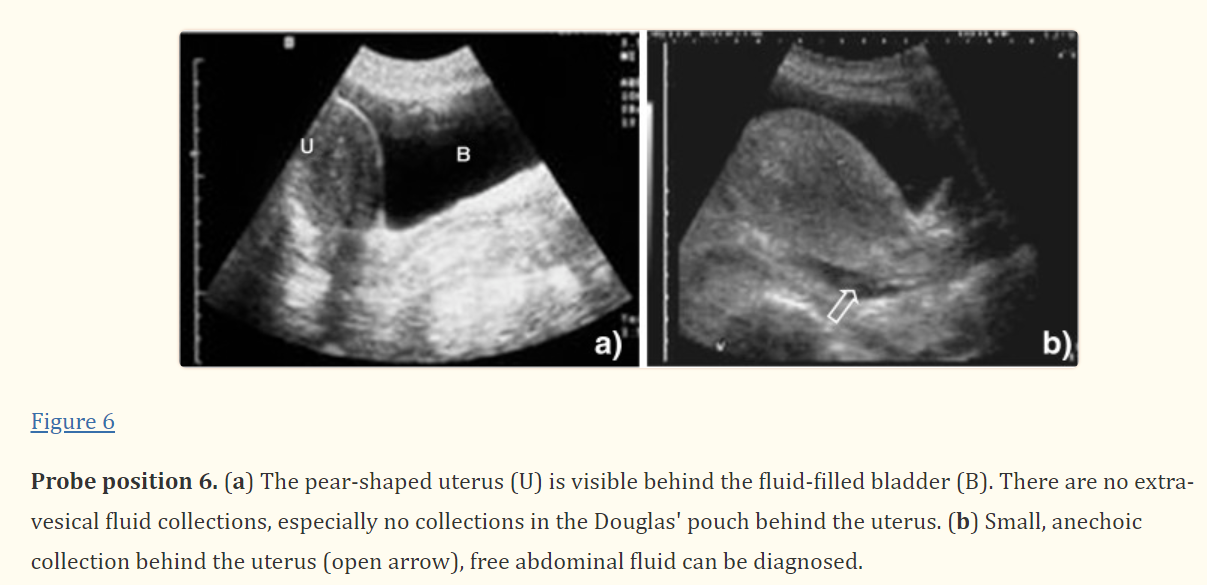


1. **Pathological findings**: Echo-free, black areas might be seen behind the bladder (male patient) or behind the uterus (female patient) in the Douglas' pouch. These represent free fluid (***Figure ​6b***).
2. **Interpretation**: Free fluid in the Douglas' pouch represents ascites. As mentioned above, other causes of ascites need to be considered beside abdominal TB. Very small amounts of fluid in the pelvis of the female patient may be normal and due to ovulation. In the female patient, pregnancies with amniotic fluid and ovarian cysts (filled with fluid) need to be considered. In comparison to free fluid, these differ in shape, are localized, and are usually limited by a wall.

## 6.5 Definitions of scan findings

- **Pericardial effusion:** Echo free space between the hyperechoic pericardium and myocardium. If present, record whether the effusion is (anechoic collection without stranding, loculations or septations) or complex (heterogenous with septations, loculations or fibrinous strands). If present, record the size.

- **Ascites:** Extraluminal echo free space in the peritoneum. If present, record whether the ascites is simple (anechoic collection without stranding, loculations or septations) or complex (heterogenous with septations, loculations or fibrinous strands).
- **Para-aortic Lymphadenopathy** is defined as the presence of hypoechoic round or ovoid structures in para-aortic region > 1.0cm confirmed to not be vascular structure by dynamic imaging techniques. If present, record the number of nodes visible in the single view sweep: 1, 2-5, >5. If present, measure the largest diameter in mm of the 2 largest visualized lymph nodes
- **Splenic Lesions** are defined as hypoechoic or hyperechoic round or ovoid lesions within the spleen. If present, record the number of lesions visible: 1, 2-5, >5
- **Hepatic Lesions** are defined as hypoechoic or hyperechoic round or ovoid lesions within the liver. If present, record the number of lesions visible: 1, 2-5, >5

## 6.6 Image acquisition

There will be an image representative of each of the views so a *total of 11 still images and 9*

*video clips*, also if there are any relevant findings, extra images will be recorded.

| **Probe position** | **Localization** | **Label** |
| --- | --- | --- |
| 1a | Epigastric angle | 1a: still image; 1a: clip |
| 1b |  | 1b: still image UA; 1b: still image PA; 1b: clip |
| 2 | Right axillary line thorax | 2:still image, 2:clip |
| 3a | Right axillary line abdomen | 3a: still image; 3a: clip |
| 3b |  | 3b: still image; 3b: clip |
| 4 | Left axillary line thorax | 4: still image; 4: clip |
| 5a | Left axillary line abdomen | 5a: still image; 5a: clip |
| 5b |  | 5b: still image; 5b: clip |
| 6 | Suprapubic pelvis | 6: still image LA; 6: still image TA; 6:clip |

dominal we call thius abdominal UStudinal orientation1xcine? i really dont know if theyre the sameng will make it complex to co

# Mediastinal lymph node ultrasound

**Protocol reference source:**

Enriquez, G., Aso, C., Serres, X. (2008). Chest Ultrasound (US). In: Lucaya, J., Strife, J.L. (eds) Pediatric Chest Imaging. Medical Radiology. Springer, Berlin, Heidelberg. <https://doi.org/10.1007/978-3-540-32676-2_1>

## 7.1 Sonographer and Patient positioning

It is recommended a small sector or convex multifrequency probes. High-frequency linear transducers are particularly useful in newborns or for studying lesions located superficially. Adjust the depth to the region of interest, decrease the number of foci, and reduce the lateral field of vision in order to increase the frame rate. Include a large vessel or cardiac chamber in the field of study to establish anatomic relationships and to analyze the echogenicity of the lesion to determine if it is cystic or solid.

The child should be placed in the supine position with a pillow or folded blanket under the shoulders and the head tilted backwards and to the left when scanning through the suprasternal notch, and in the left lateral position when using the parasternal approach.


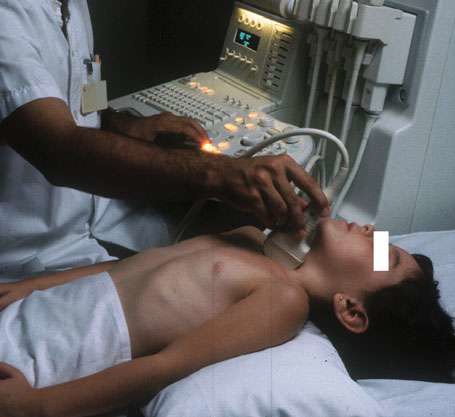

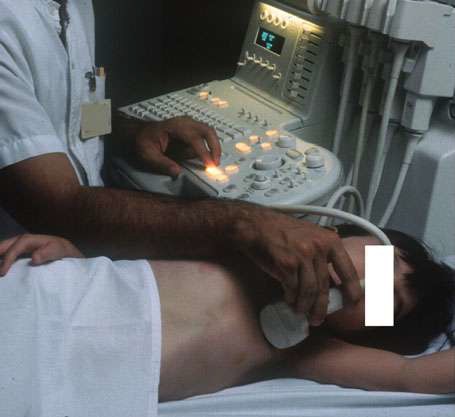


***Figure: Patient positioning for US access to the mediastinum using the suprasternal (a) and left parasternal (b) approaches***

## 7.2 Regions covered

The mediastinum is divided into anterior, middle and posterior compartments:


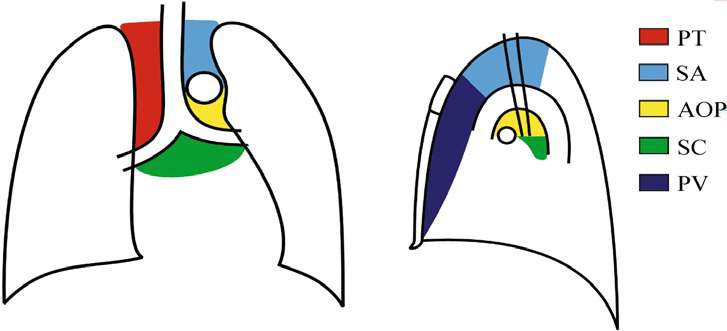


***Figure . Diagrams showing US division of the anterior and middle radiologic mediastinal regions: PV, prevascular; PT, paratracheal; SA, supraaortic; AOP, aortopulmonary; SC, subcarinal.***

1. **The anterior mediastinum** – also known as the prevascular region is in front of the superior vena cava, aorta and pulmonary artery and behind the sternum.
2. **The middle mediastinum** – further divided into 4 regions (paratracheal, supra-aortic, aortopulmonary and subcarinal):
   1. ***Paratracheal region*** – right paratracheal area
   2. ***Supra-aortic*** – upper portion of the left paratracheal region, above the aortic arch
   3. ***The aortopulmonary*** – the area below the aortic arch and above the right pulmonary artery and the left bronchus
   4. ***The subcarinal*** – behind the bifurcation of the pulmonary artery, above the left atrium in front of the esophagus and below the carina.
3. **The posterior mediastinum** – the pre- and paravertebral spaces.

## 7.3 Definition of scan findings

**Mediastinal lymph nodes** are defined as hypoechoic oval structures located within the mediastinum that may displace surrounding structures such as vessels and airways.

## 7.4 General approach and specific approach

The anterior and middle mediastinum can be well accessed with the ***suprasternal and the left parasternal approaches***. Additional approaches which can be used are supraclavicular, subxiphoid, and subcostal.

Five standard sonographic slices are needed to visualize the complete anterior and middle regions of the mediastinum: three obtained with the suprasternal approach and two with the left parasternal.

**Through the suprasternal notch**

The child is positioned as described in 7.1. and access to the mediastinal window with the potential to identify lymph nodes is achieved using 3 views:

| **M1. Oblique parasagital view.** | 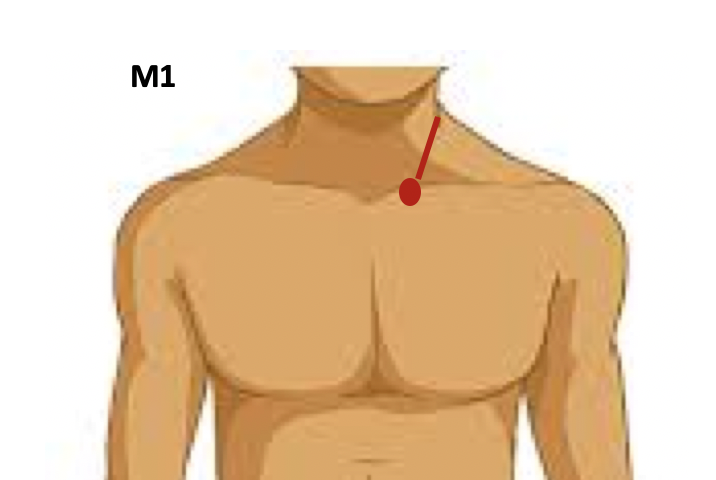 |
| --- | --- |
| Oblique parasagittal view through the suprasternal approach is shown in Figure below. This view is used to visualize the aortopulmonary region, which has a characteristic ultrasound appearance and should be identiﬁed in all patients submitted to mediastinal exams. The probe is placed above the sternal manubrium between the trachea and left sternocleidomastoid muscle. Due to the presence of mediastinal fat at this level, the aortopulmonary region is seen as a highly echogenic, half-moon- shaped image. The anatomic reference for this region is the aortic arch and the section should also include the origins of the left carotid and subclavian arteries. | |
| 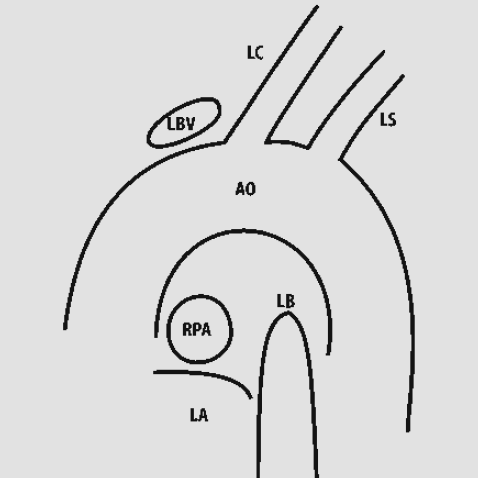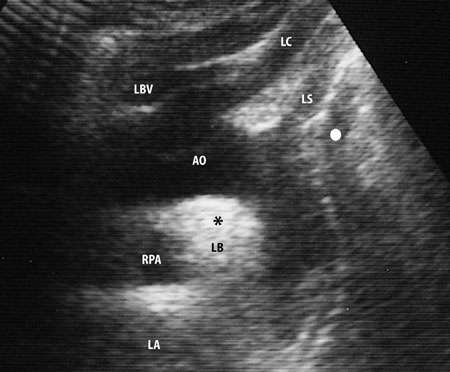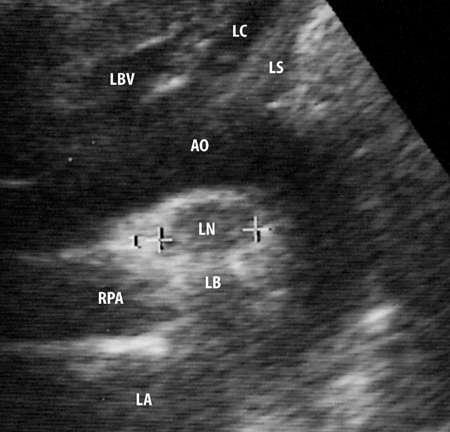 | |

| **M2. Oblique Coronal view:** | 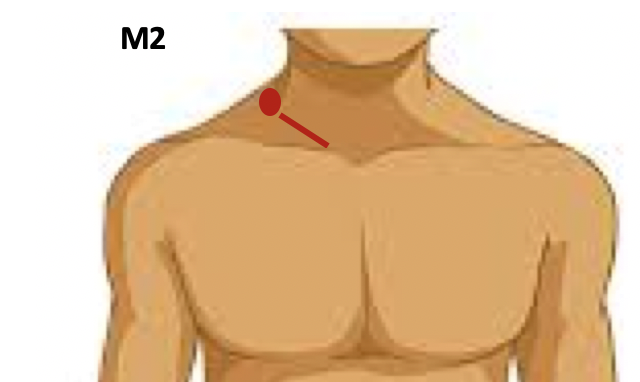 |
| --- | --- |
| This section is used to visualize the paratracheal region, located between the right upper pulmonary lobe and the trachea. It is also useful for studying the aortopulmonary region, which is seen in this view as an echogenic triangular image. The probe is placed in the transverse position in the suprasternal notch and is angled downwards towards the chest.  The probe is placed almost perpendicular to, and slightly compressing, the right sternocleidomastoid muscle. The anatomic reference for this region is the innominate artery and the scan should include the pleural surface of the right upper lobe, the trachea and the upper margin of the left bronchus. The paratracheal region is a virtual space and is considered normal when the pleural surface of the upper lobe abuts the trachea. Pathology in this region is recognized by a separation of these two structures | |
| 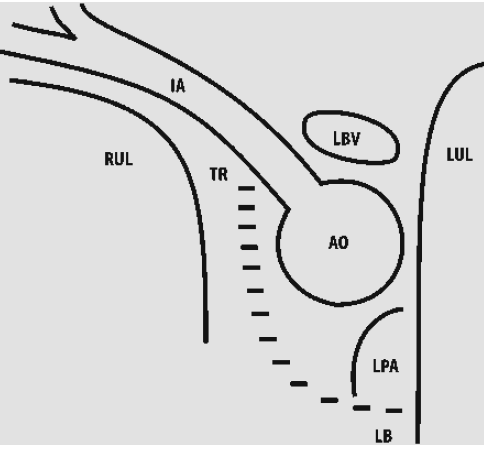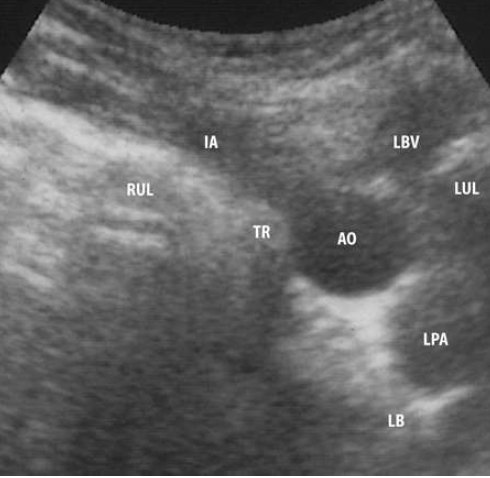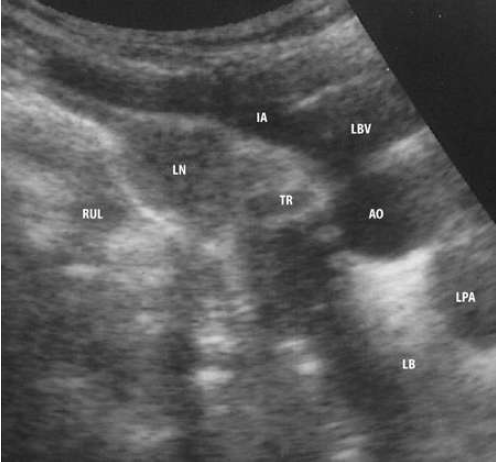 | |

| **M3. Coronal view.** | 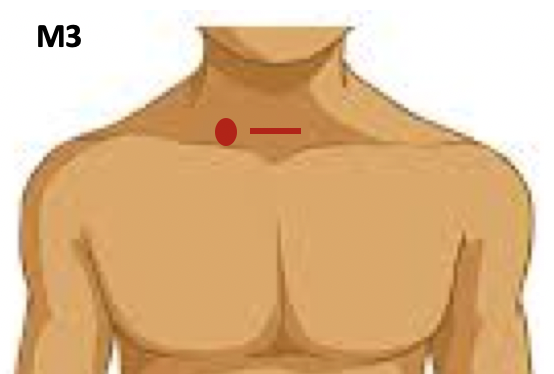 |
| --- | --- |
| Coronal view through the suprasternal approach is shown in Figure below. This scan is not used to study a specific mediastinal region, but is very useful for visualizing the vessels, particularly the superior vena cava. The view should include the right pulmonary artery and its bifurcation, located within the mediastinum | |
| 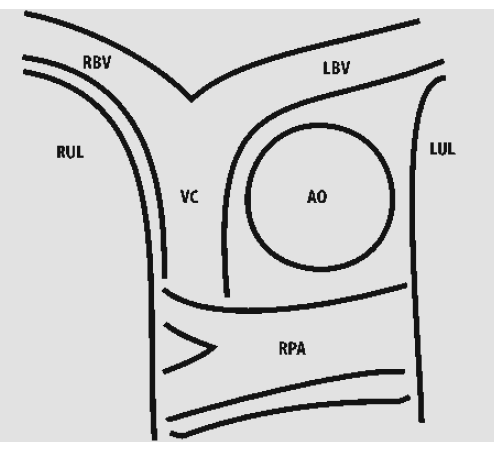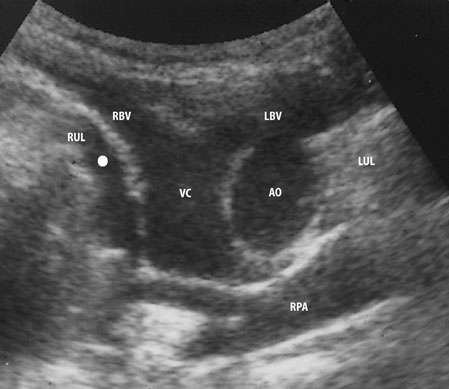 | |

**Parasternal approach:**

The child is placed in the left lateral position (the examined side down) is recommended to displace the mediastinum downwards and increase the acoustic window and the US probe is placed in the intercostal spaces alongside the sternum.

| ***M4. Axial view on parasternal approach*** | 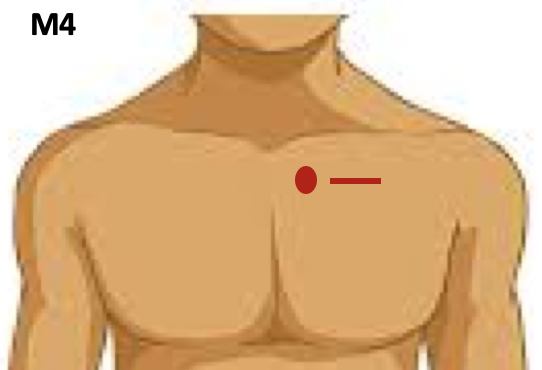 |
| --- | --- |
| This section is used to study the subcarinal and pre-vascular regions and is similar to the CT slices obtained for this area. The transducer is placed at the second intercostal space. The anatomic reference for this view is the pulmonary artery bifurcation (Figure below). | |
| 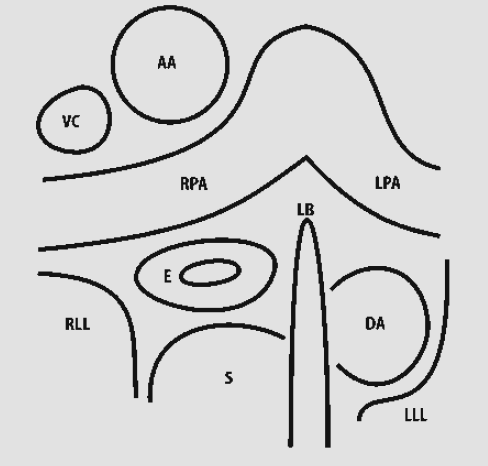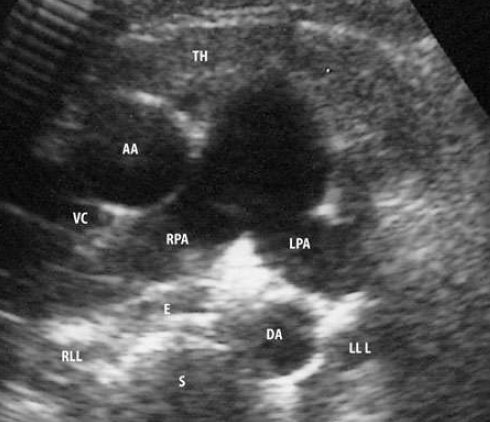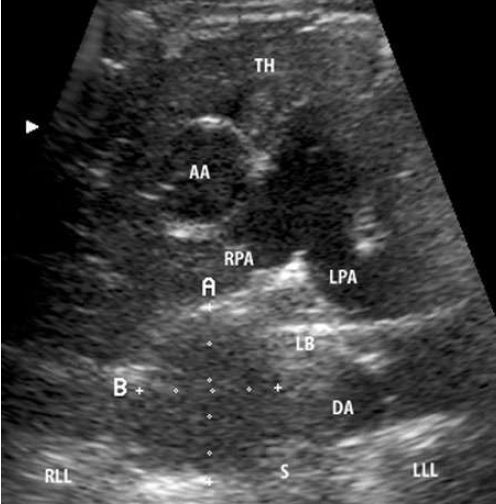 | |

| ***M5. Parasagittal view through the left parasternal approach*** | 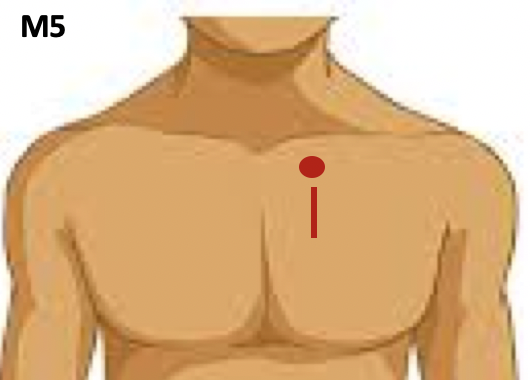 |
| --- | --- |
| Also used to study the subcarinal and pre-vascular regions, the references for this section are the ascending aorta, the trachea and the esophagus. It is important to identify the esophagus by making the patient swallow saliva or water. For the two parasternal approaches, the patients should be placed in a left decubitus position to increase the size of the anatomic acoustic window. | |
| 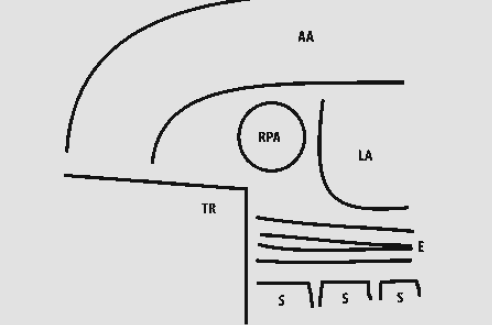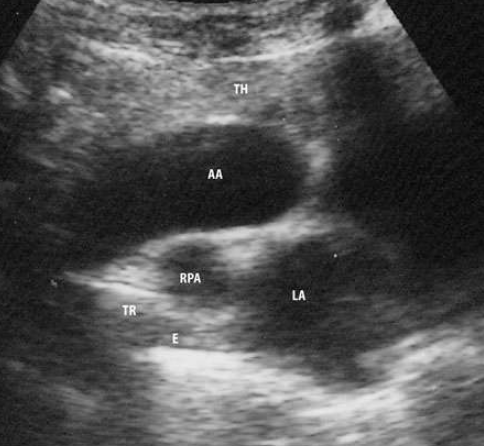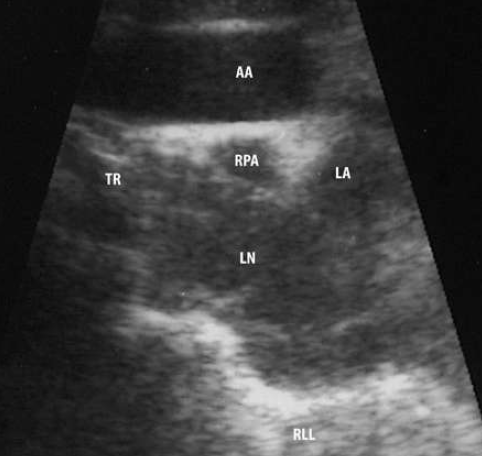 | |

## 7.5 Definitions of scan findings

1. **Thymus** – the normal thymus has a bilobulated appearance and a homogeneous echotexture with some echogenic strands. It is hypoechoic relative to the thyroid gland and has a smooth, well defined margin due to its fibrous capsule. It is a soft organ that does not compress neighbouring vascular structures, a characteristic that can help the radiologist to differentiate it from mediastinal masses.
2. **Lymph nodes** – scattered or clustered nodules with varying degrees of echogenicity. The right paratracheal region, pulmonary hilum and subcarinal space are the most common sites of pathologic lymph nodes. The US appearance of tuberculous nodes can change following treatment and present a hypoechoic center and echogenic halo, probably due to internal caseous material.

## 7.6 Image acquisition

A still image and 1 video (5 seconds) clip of each of the “views” (M1 to M5) will be taken once all the recognizable structures are visualized on the screen – ***a total of 5 still images and 4 video clips (5sec)***. Additionally, if there are any pathological findings more than 1 image can be taken to accurately represent the finding.

| **Probe position** | **View** | **Localization** | **Label** |
| --- | --- | --- | --- |
| M1 | Oblique parasagittal | Suprasternal notch | M1: still image; M1: clip |
| M2 | Oblique coronal | Paratracheal | M2: still image; M2: clip |
| M3 | Coronal | Suprasternal notch | M3: still image; M3: clip |
| M4 | Axial | Parasternal | M4: still image; M4: clip |
| M5 | Parasagittal | Left parasternal | M5: still image; M5: clip |

# 8. Assessing scan quality and other general assessments

## 8.1 Quality

For each region as assessment will be made as to whether the quality of the scan was:

- - - **1** = No recognizable structures. No objective data can be gathered;
    - **2** = Minimally recognizable structures. Insufficient for diagnosis;
    - **3** = Minimal criteria met for diagnosis. Recognizable structures, some technical or other flaws;
    - **4** = Minimal criteria met for diagnosis. All structures imaged well. Diagnosis easily supported;
    - **5** = Minimal criteria met for diagnosis. All structures imaged with excellent image quality. Diagnosis completely supported

## 8.2. Other

1. Write down the name (initials) /code of sonographer performing the exam and whether they are blinded to the relevant clinical information at the time of scan (including previous imaging exams, patient´s sign/symptoms, bacteriological confirmation etc).
2. Record, for each of the three approaches separately, the time of scan start/finish, the overall evaluation (normal/abnormal, and suggestive /not suggestive of TB) and whether the scan had an impact in TB diagnostic decision making.
3. Rate the patient cooperation from 1 to 5 (1 being worse possible and 5 being best).

# Completing the US exams and documenting the findings

Ensure that all cine-loops and images have been appropriately labelled and saved:

- - - Ensure that the ultrasound exam correctly saved on the PACS system
    - Study team to still define safe mode of uploading images to a central study server.

Complete the “US CRF” digitally on the tablet or in paper format

- - - No fields should be left blank
    - Assign study number (Hospital number_consecutive number e.g. 2_001)
      - - H12O= 1
        - HGM= 2
        - HVH= 3
        - HSJD= 4
    - Ensure that the data is saved and ‘sent to server’ after capturing.
