# Supplementary material for: Diagnostic Performance of Comprehensive Point‐of‐Care Ultrasound for Pediatric Tuberculosis in Spain: A Prospective Observational Study
Source: J Clin Ultrasound. 2026 Mar 15;54(6):1425–32. doi: 10.1002/jcu.70228 (PMC13332548; doi:10.1002/jcu.70228)
Supplement: Supplementary file 2 — Table S1: Completeness of cPOCUS exams by TB category (n = 27). Table S2: Summary of main findings across all imaging modalities. Table S3: Imaging findings among children with TB disease only (n = 12). Table S4: Side‐by‐side comparison of TB vs non‐TB cases. Table S5: Comparison of radiologist vs expert cPOCUS findings by anatomical compartment. [file JCU-54-1425-s002.docx]

**Supplementary material 2**

**Supplementary table 1: Completeness of cPOCUS exams by TB category (n = 27)**

| **Case number** | **TB Category** | **POCUS Exam Completion (Expert Assessment)** |
| --- | --- | --- |
| 1 | TB | Incomplete: mediastinum done; no lung or abdominal US |
| 2 | TB | Complete |
| 3 | TB | Complete |
| 4 | NonTB | Incomplete: mediastinum incomplete; lung not all regions |
| 5 | NonTB | Incomplete: mediastinum incomplete; lung not all regions |
| 6 | TB | Incomplete: mediastinum incomplete; no lung US |
| 7 | NonTB | Incomplete: mediastinum incomplete |
| 8 | NonTB | Incomplete: lung US not complete |
| 9 | NonTB | Incomplete: lung US not complete |
| 10 | NonTB | Incomplete: lung US not complete |
| 11 | NonTB | Incomplete: lung US not complete |
| 12 | TB | Incomplete: lung US not complete |
| 13 | TB | Incomplete: lung US not complete |
| 14 | NonTB | Incomplete: mediastinum, lung, abdomen done but not all regions |
| 15 | NonTB | Incomplete: mediastinum, lung, abdomen done but not all regions |
| 16 | NonTB | Incomplete: no mediastinum; lung + abdomen done but not all regions |
| 17 | NonTB | Incomplete: mediastinum, lung, abdomen done but not all regions |
| 18 | NonTB | Incomplete: mediastinum, lung, abdomen done but not all regions |
| 19 | TB | Incomplete: no mediastinum US done |
| 20 | TB | Complete |
| 21 | TB | Incomplete: mediastinum US not done |
| 22 | TB | Incomplete: no abdominal US done |
| 23 | NonTB | Complete |
| 24 | TB | Complete |
| 25 | NonTB | Complete |
| 26 | TB | No US done |
| 27 | NonTB | Complete |

**Supplementary table 2: Summary of main findings across all imaging modalities**

|  | **Findings** |
| --- | --- |
| **Imaging completion** | - CXR: 27/27 - cPOCUS radiologist: 27/27 - cPOCUS expert: 26/27 - CT: 14/27 |
| **CXR pathologies** | Abnormal 13/27 (48.1%). Most frequent:   - hilar adenopathy (7) - paratracheal/subcarinal adenopathy (5) - parenchymal opacities (4) - airway stenosis/atelectasis (1) - nodule (1) - pneumatocele (1). |
| **CT pathologies** | Abnormal 9/14 (64.3%). Most frequent:   - multi-station lymphadenopathy (hilar, paratracheal, subcarinal) - airway compression/stenosis - atelectasis, opacities. |
| **Radiologist cPOCUS pathologies*** | Abnormal 9/27 (33.3%). Main findings:   - AOP/paratracheal lymph nodes (5) - small subpleural/lung consolidations (3) - abdominal lymph nodes (3). |
| **Expert cPOCUS pathologies*†** | Abnormal 10/26 (38.5%). Most frequent:   - enlarged AOP window nodes >1 cm (4) - lung consolidations (4) - mesenteric/peripheral nodes (3). |
| **Most frequent pathology overall** | Mediastinal lymphadenopathy across CXR, CT, and cPOCUS; CT showed most stations, cPOCUS excelled at AOP window nodes. |

** Abnormal = at least one of mediastinum / lung / abdomen rated “Pathological”. †n with interpretable cPOCUS: 26/27 (1 child with all key fields missing due to “no US done” categories)*

**Supplementary table 3: Imaging findings among children with TB disease only (n = 12)**

|  | **Findings in TB cases** |
| --- | --- |
| **Imaging completion** | All 12 underwent CXR and cPOCUS; CT performed in 9/12. |
| **CXR abnormalities** | 9/12 (75.0%) abnormal. Dominant findings: hilar and paratracheal lymphadenopathy (6), parenchymal opacities (3), airway stenosis/atelectasis (1). Mediastinal lymphadenopathy was the dominant pattern. |
| **CT abnormalities** | 7/9 (77.8%) abnormal. Most common: multi-station lymphadenopathy (hilar/paratracheal/subcarinal), airway compression/stenosis, atelectasis, parenchymal opacities. CT consistently identified more extensive nodal disease than CXR or cPOCUS. |
| **Radiologist cPOCUS abnormalities** | 7/12 (58.3%) abnormal. Frequent findings: AOP/paratracheal lymph nodes (4), small subpleural or focal consolidations (2), abdominal lymph nodes (2). |
| **Expert cPOCUS abnormalities** | 7/11 (63.6%) abnormal. Frequent findings: AOP window lymph nodes >1 cm (4), lung consolidations (3), mesenteric nodes (2). Experts detected slightly more subtle mediastinal and pulmonary abnormalities. |
| **Most frequent pathology across modalities** | Mediastinal lymphadenopathy was universally the most common abnormality across CXR, CT, and cPOCUS. CT identified the largest number of lymph node stations; cPOCUS excelled at detecting AOP window lymph nodes. |
| **CXR-CT-cPOCUS concordance** | CXR and CT aligned in identifying multi-station lymphadenopathy in most TB cases. cPOCUS findings (both readers) corresponded with CT in cases with AOP lymphadenopathy. Radiologist and expert cPOCUS showed near-identical patterns, with experts identifying more subtle abnormalities. |

**Supplementary table 4: Side-by-side comparison of TB vs non-TB cases**

|  | **TB cases (n = 12)** | **Non-TB cases (n = 15)** |
| --- | --- | --- |
| **Imaging completion** | CXR: 12/12; CT: 9/12; cPOCUS radiologist & expert: 12/12 | CXR: 15/15; CT: 5/15; cPOCUS radiologist: 15/15; expert: 15/15 |
| **CXR abnormalities** | 9/12 (75.0%) abnormal; predominately LN disease | 4/15 (26.7%) abnormal; isolated nodes or non-specific opacities |
| **CT abnormalities** | 7/9 (77.8%); extensive multi-station LN disease, airway changes | 2/5 (40.0%); mild or incidental findings |
| **Radiologist cPOCUS abnormalities** | 7/12 (58.3%); AOP nodes and consolidations | 2/15 (13.3%); mostly normal or physiological nodes |
| **Expert cPOCUS abnormalities** | 7/11 (63.6%); enlarged AOP nodes and lung consolidation | 3/15 (20.0%); small or normal-range nodes |
| **Dominant pathology** | Mediastinal lymphadenopathy; parenchymal consolidations | Absence of significant lymphadenopathy; isolated small nodes |
| **CXR vs cPOCUS (TB)** | Both detect LN disease; cPOCUS finds AOP nodes earlier | CXR and cPOCUS often normal |
| **CT vs cPOCUS (TB)** | CT shows deeper LN disease; cPOCUS detects accessible AOP nodes | Limited CT - cPOCUS correlation due to few abnormalities |
| **Radiologist vs expert cPOCUS** | High concordance; experts detect more subtle abnormalities | High concordance; both mostly normal |
| **Overall interpretation** | Strong radiological evidence of LN-dominant TB across modalities | Normal or near-normal imaging; lymphadenopathy uncommon |

*LN: lymph node*

**Supplementary table 5: Comparison of radiologist vs expert cPOCUS findings by anatomical compartment**

| **Compartment** | **Radiologist: abnormal (n)** | **Expert: abnormal (n)** | **Agreement** | **Key differences** |
| --- | --- | --- | --- | --- |
| **Mediastinum** | 5 | 7 | High | Experts identified additional AOP window nodes and one latero-cervical node not flagged by radiologists. |
| **Lung** | 3 | 4 | High | Experts detected one additional small focal consolidation (L2 – L4). |
| **Abdomen** | 3 | 3 | Very high | Both detected similar mesenteric/periportal nodes; no major discrepancies. |
